# Old African fossils provide new evidence for the origin of the American crocodiles

**DOI:** 10.1038/s41598-020-68482-5

**Published:** 2020-07-23

**Authors:** Massimo Delfino, Dawid A. Iurino, Bruno Mercurio, Paolo Piras, Lorenzo Rook, Raffaele Sardella

**Affiliations:** 1 Dipartimento di Scienze della Terra, Università di Torino, Via Valperga Caluso 35, 10125 Turin, Italy; 2 Institut Català de Paleontologia Miquel Crusafont, Universitat Autònoma de Barcelona, Edifici ICTA-ICP, Carrer de les Columnes s/n, Campus de la UAB, 08193 Cerdanyola del Vallès, Barcelona Spain; 3 PaleoFactory, Dipartimento di Scienze della Terra, Sapienza Università di Roma, Piazzale Aldo Moro 5, 00185 Roma, Italy; 4 Dipartimento di Fisica e Geologia, Università degli Studi di Perugia, Via A. Pascoli, 06123 Perugia, Italy; 5 Dipartimento di Ingegneria Strutturale e Geotecnica, Sapienza Università di Roma, 00184 Roma, Italy; 6 Center for Evolutionary Ecology, Largo S. Leonardo Murialdo 1, 00146 Roma, Italy; 7 Dipartimento di Scienze della Terra, Paleo[Fab]Lab, Università di Firenze, Via G. La Pira 4, 50121 Florence, Italy; 8 Dipartimento di Scienze della Terra, Sapienza Università di Roma, Piazzale Aldo Moro 5, 00185 Roma, Italy

**Keywords:** Palaeontology, Zoology, Biogeography

## Abstract

Molecular and morphological phylogenies concur in indicating that the African lineages formerly referred to *Crocodylus niloticus* are the sister taxon the four Neotropical crocodiles (*Crocodylus intermedius*, *C. moreleti*, *C. acutus* and *C. rhombifer*), implying a transoceanic dispersal from Africa to America. So far the fossil record did not contribute to identify a possible African forerunner of the Neotropical species but, curiously, the oldest remains referred to the African *C. niloticus* are Quaternary in age, whereas the oldest American fossils of *Crocodylus* are older, being dated to the early Pliocene, suggesting that another species could be involved. We re-described, also thanks to CT imaging, the only well-preserved topotipic skull of *Crocodylus checchiai* Maccagno, 1947 from the late Miocene (Messinian) African site of As Sahabi in Libya. As previously suggested on the basis of late Miocene material from Tanzania, *C. checchiai* is a valid, diagnosable species. According to our phylogenetic analyses, *C. checchiai* is related to the Neotropical taxa and could be even located at the base of their radiation, therefore representing the missing link between the African and the American lineages.

## Introduction

Extant crocodylians are represented by 25 species grouped into 9 genera^1^. The most speciose and widespread genus is *Crocodylus* Laurenti, 1768 that hosts 12 species inhabiting a longitudinally very broad intertropical belt ranging from Australia to South Asia, to Africa and then America. The origin of *Crocodylus* was placed in the late Miocene (about 13.6–8.3 Ma) of Asia by Oaks^2^, on the basis of a time-calibrated species tree stemming from a DNA analysis, and recently reconstructed as Asian by Nicolai and Mazke^3^ on the basis of historical biogeography models. Both the molecular^2,4,5^ and the morphological/palaeontological^6–9^ approaches, as well as the shared presence of parasites^10^ concur in indicating that the origin of the American clade of *Crocodylus* should be sought in Africa. The four American species *Crocodylus intermedius*, *C. moreleti*, *C. acutus* and *C. rhombifer*are grouped together with the African *Crocodylus niloticus* Laurenti, 1768, which is placed at the base of the American branch. According to Meredith et al.^5^, the most parsimonious explanation that takes into consideration both the phylogenetic results and the fossil record of *Crocodylus* supports a relatively recent trans-Atlantic crossing of “*C. niloticus*” from Africa to America. The reason of the inverted commas placed by these authors around the name of Nile crocodile is related to the fact that the eastern and western clades of this species do not cluster together, but are arranged in paraphyly at the base of the American clade, a result shared by different authors applying different molecular techniques^2,4,5,11,12^. Hekkala et al.^5^ eventually proposed the resurrection of the name *Crocodylus suchus* Geoffroy Saint-Hilaire, 1807 for the western populations, keeping the name *C. niloticus* for the (predominantly) eastern populations that is directly at the base of the American clade. Probably because of the dominance of the molecular approach involved in the analysis of the phylogenetic relationships and biogeographic history of extant taxa, the African fossil record has been only marginally involved in the discussion concerning the origin of the American clade^6,7,8,9^, even if Hect^13^ already underlined in 1987 that an African extinct species shared with the American clade a synapomorphic trait.

### *Crocodylus checchiai* Maccagno, 1947

**Figure 1:**
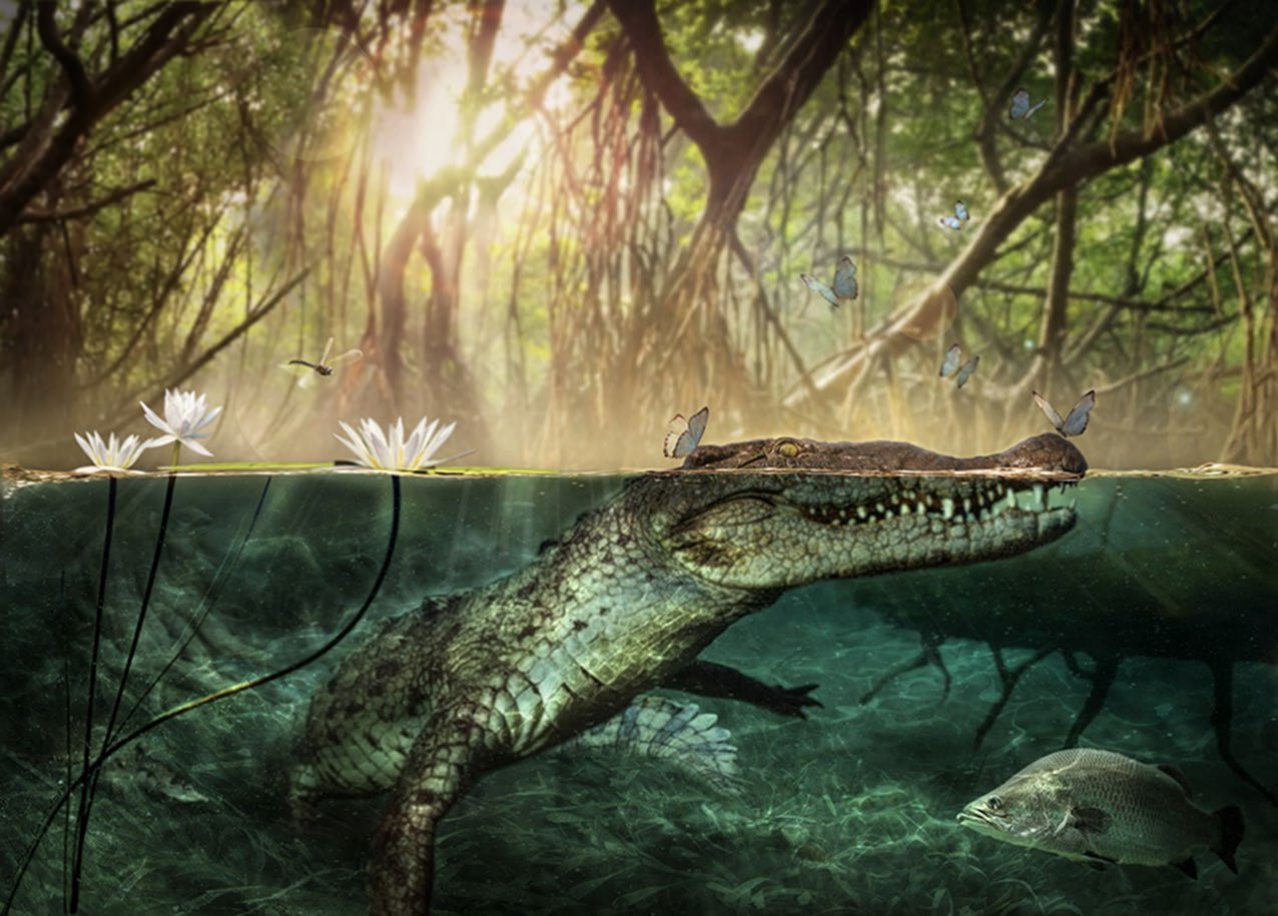
Life appearance of *Crocodylus checchiai* from As Sahabi (Libya). The reconstruction is based on the information from literature^13–15^ updated with the complete cranium sn813/lj. Artwork by D. A. Iurino.

**Figure 2:**
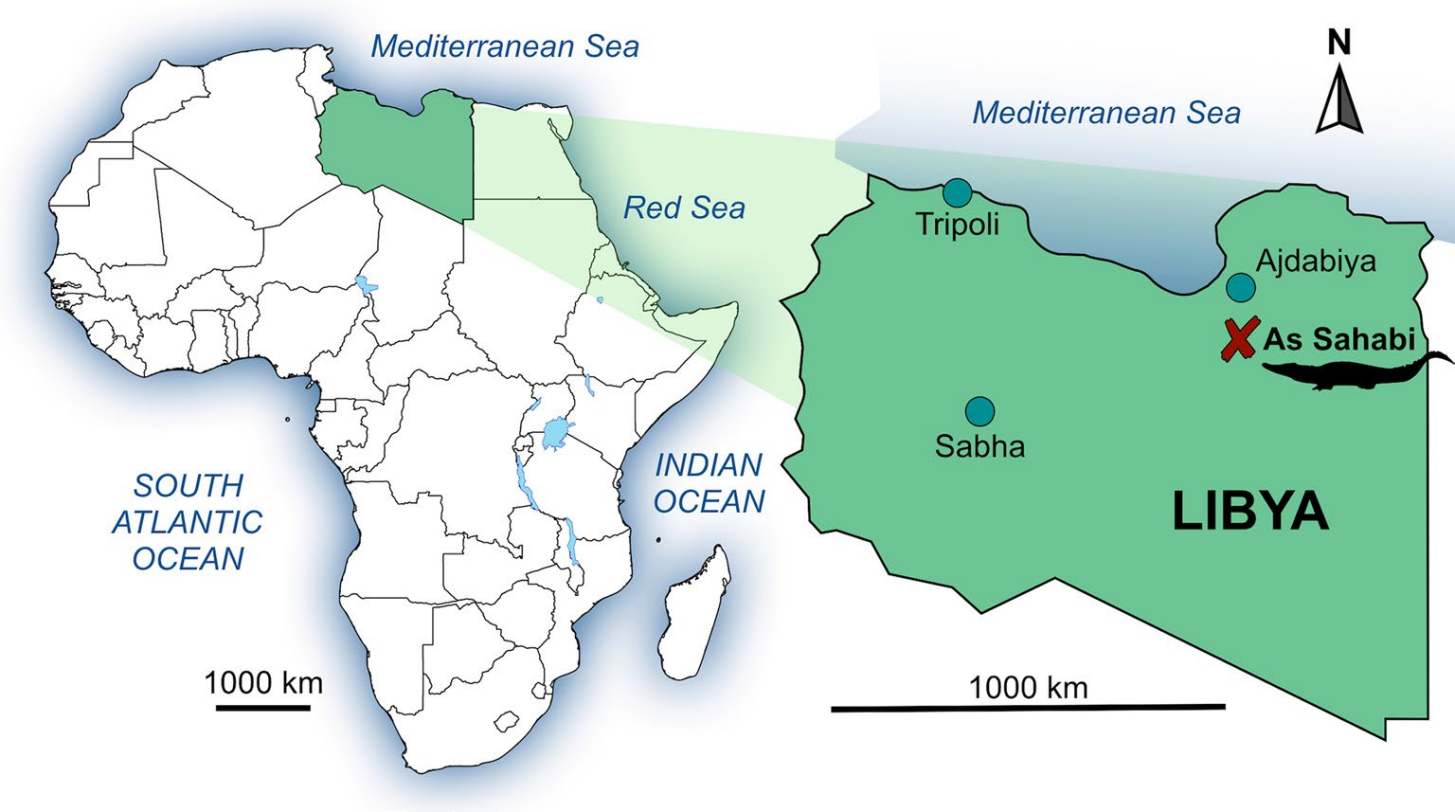
Geographic location of the As Sahabi paleontological locality. Artwork by D. A. Iurino.

This species (Fig. 1) was originally described in Italian by Maccagno^14^ on the basis of an adult, well-preserved skull and associated lower jaw collected in 1938 by Petrocchi in the area of As Sahabi in Libya and then hosted in the collections of the Istituto di Paleontologia dell’Università di Roma (now apparently lost). As Sahabi is a celebrated latest Miocene vertebrate locality in northern Libya, located about 130 km south of Ajdabiya along the road going south into the Libyan Sahara to Gialo and Kufra (Fig. 2). The discovery of the paleontological site and the recovery of abundant fossils at As Sahabi in the 1930′s is due to the invaluable efforts of C. Petrocchi^16,17^.

Maccagno provided a thorough description of the skull and a lengthy series of comparisons with some of the extinct and extant crocodylian taxa known at that time, comparisons that are now of little value in the context of the currently known phylogenetic relationships. However, among the several characters listed as diagnostic for *C. checchiai* at least two are still considered of taxonomic and phylogenetic relevance: the marked median gibbosity located in the posterior sector of the nasals (involving also the maxillae and the lacrimals), as well as the anterior tip of the nasals entering the narial opening.

A few years later, in a second paper on the crocodiles from As Sahabi, Maccagno^15^ reported the presence of four more skulls. The three skulls (one with lower jaw) stored in the Museum of Natural History of Tripoli were destroyed or lost during the II World War, but she published their photograph in lateral view^15^. The fourth skull, collected by Petrocchi in 1939 and brought to Rome was described by Maccagno^15^ and referred to the new variety, *Crocodylus checchiai* var. *depressa* characterized by slightly broader posterior region of the skull. Few years ago, fragmentary new material from the same Libyan area of As Sahabi (30P24A belonging to the University of Garyounis Earth Science Museum, but currently on loan at the Department of Earth Sciences of the University of Florence, Italy) was referred to *C. checchiai* by Delfino^18^ without any phylogenetic consideration. Later Brochu and Storrs^9^ referred two skulls (KNM-LT 23108 and KNM-LT 26618) from the late Miocene–early Pliocene Nawata Formation at Lothagam (Tanzania) to the same taxon. On the basis of the Tanzanian remains, they included for the first time *C. checchiai* in a phylogenetic context and concluded that this species cannot be excluded from *Crocodylus*, but retrieved a broad politomy that did not support any precise relationship.

In order to improve the knowledge about *C. checchiai* and its possible relationships with the Neotropical crocodile species, we tried to locate the original material described by Maccagno and succeeded in finding in the collection of the MUST (Museo Universitario di Scienze della Terra) of Sapienza University of Rome, the skull sn813/lj on which Maccagno^15^ based the taxon *C. checchiai* var. *depressa*. We here redescribe the specimen also on the basis of tomographic imaging that allow us to study the inner structures, taking into consideration the characters currently considered taxonomically diagnostic and phylogenetically relevant. The phylogenetic relationships were explored thanks to the character matrix by Brochu and Storrs^9^ that includes several African crocodylines, as well as on the basis of that of Scheyer et al.^19^ that includes the geological oldest American *Crocodylus*, e.g. *C. falconensis*.

## Systematic palaeontology

- Crocodylia Gmelin, 1789, sensu Benton and Clark, 1988.
- Crocodylidae Cuvier, 1807, sensu Brochu, 2003.
- *Crocodylus* Laurenti, 1769.
- ***Crocodylus checchiai*** Maccagno, 1947.

### Institutional abbreviations

**KNM**, National Museums of Kenya, Nairobi, Kenya; **MPURLS**, Museo di Paleontologia dell’Università di Roma La Sapienza, Rome, Italy; **MUST**, Museo Universitario di Scienze della Terra, Sapienza University of Rome, Italy; **sn**, senza numero d’inventario (temporary inventory number).

### Referred material

**Figure 3:**
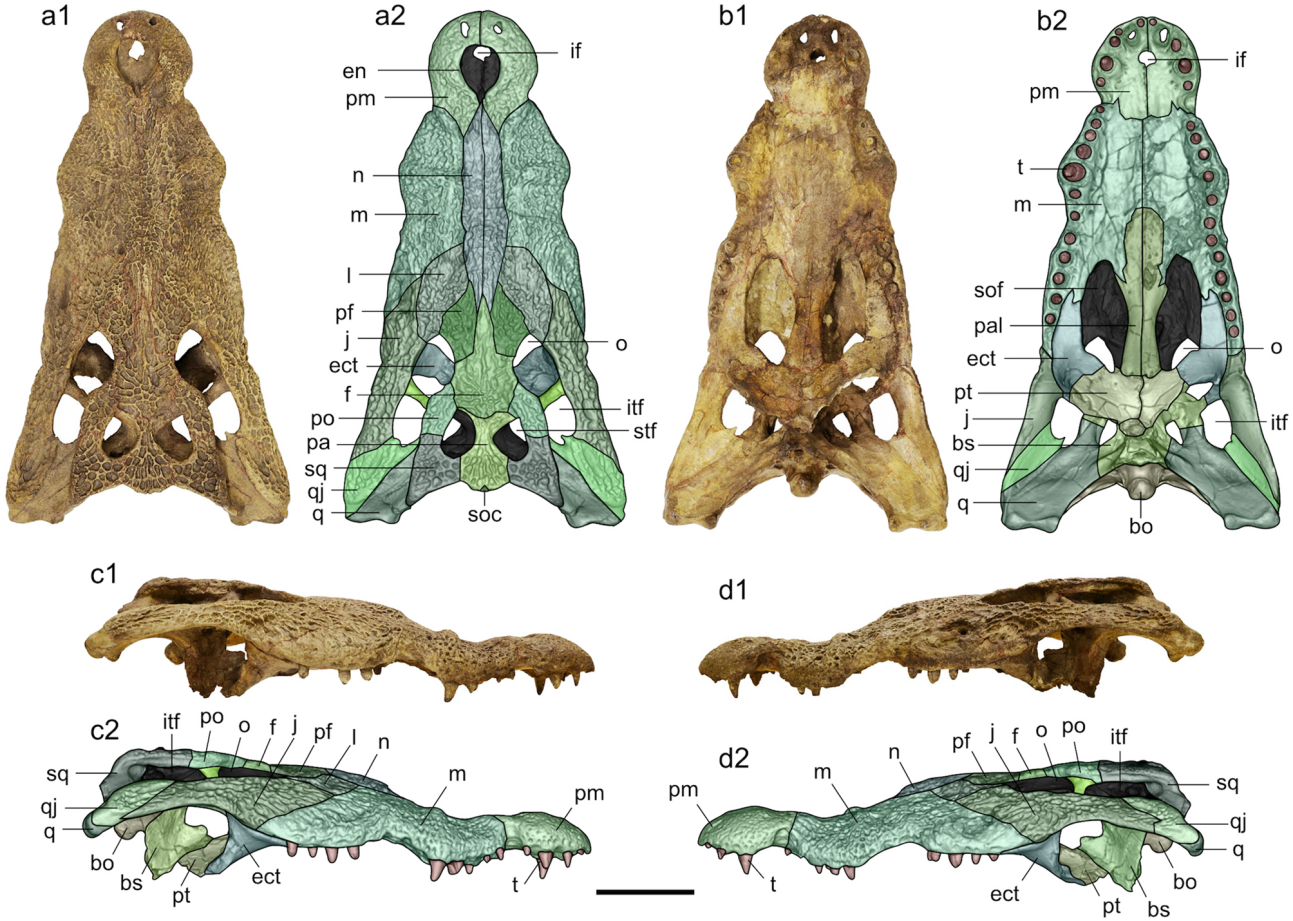
*Crocodylus checchiai*^14^ from As Sahabi. Specimen sn813/lj in dorsal (**a1**,**a2**), ventral (**b1**,**b2**), right lateral (**c1**,**c2**) and left lateral (**d1**,**d2**) views. Anatomical abbreviations: bo, basioccipital; bs, basisphenoid; ect, ectopterygoid; en, external naris; f, frontal; if, incisive foramen; itf, infratemporal fenestra; j, jugal; l, lacrimal; m, maxilla; n, nasal; o, orbit; pa, parietal; pal, palatine; pf, prefrontal; pm, premaxilla; po, postorbital; pt, pterygoid; q, quadrate; qj, quadratojugal; soc, supraoccipital; sof, suborbital fenestra; sq, squamosal; stf, supratemporal fenestra; t, tooth. Scale bar: 10 cm.

Cranium sn813/lj (ex MPURLS) (Fig. 3, Supplementary Video S1) stored at MUST.

### Occurrence

Albeit the age of the As Sahabi vertebrate fauna has long been debated, we concur with most recent studies^20^ in which the geological context suggests a Messinian age, around 7 Ma, for As Sahabi vertebrate bearing levels. According to Bernor and Rook^21^ and Bernor et al.^22^ in respect to faunal biogeography and biochronology, As Sahabi is best correlated with the Upper Nawata of Lothagam (Kenya) and MN13 Mammal Unit in the European Neogene Mammal biochronological time scale^23^.

### Emended diagnosis

*Crocodylus checchiai* differs from all the other extant or extinct *Crocodylus* species because of the combination of the following characters: presence of medial rostral boss (the only African species so far described with a medial rostral boss); broad contact of lacrimal with nasal without a posterior process of the maxilla; flattened (or nearly so) posterolateral margin of the squamosal; quadratojugal extension to superior angle of infratemporal fenestra. Noteworthy is that *C. checchiai* is characterized by 5 premaxillary and 13 maxillary alveoli. According to Maccagno^14^ this species has 12 dentary alveoli.

## Description

### Preservation and general features

The specimen sn813/lj (Fig. 3, Supplementary Video S1) is a relatively well-preserved, minimally distorted mesorostral skull, whose dorsal length from the preserved anterior tip of the premaxillae to posterior tip of the supraoccipital corresponds to 47.0 cm. Pterygoids and basisphenoid are incomplete. The anterior tip of the nasals is missing. A small lateral area on the left maxilla, at the level of the posterior most alveoli, is damaged. The dorsal surface of the skull hosts a complex pattern of pits and ridges that build up a dense and evident ornamentation. Most of the surface is however covered by a thin layer of arenaceous crusts that hinders some of the sutures and fine morphological details. The rostrum shows a marked festooning in both dorsal and lateral views. The lateral surface of the snout is distinctly constricted in dorsal view at the level of the premaxillary-maxillary suture. The slight asymmetry visible in dorsal view is even more evident on the ventral surface, where, although there is a distinct lateral notch on both sides, the (anatomically) right one is better defined. On the right side there are two low longitudinal ridges delimiting, dorsally and ventrally, an elongated, lateral shallow concavity. A distinct convexity develops on the dorsal surface of both maxillae in correspondence of the root of the fifth maxillary tooth. The most striking character of the rostrum is the presence of a mid-rostral boss that involves anteriorly the posterior area of the nasals, but also the prefrontals, lacrimals (where a sort of accessory bump is present) and the anterior tip of the frontal process posteriorly. Canthi rostralii are absent, as well as marked preorbital ridges and antorbital fenestrae. The trapezoidal skull table is relatively small and flat (slightly concave in posterior view if the small bump of the right squamosal is considered), but a breakage, followed by partial displacement, lowered the area anterior to the right supratemporal fenestra.

### Fenestrae and major openings

The external naris opens flush with dorsal surface of premaxillae and is slightly anterposteriorly elongated, but not prominently teardrop-shaped (therefore character 83 was scored as 0). Its posterior rim is broken off for about 1 cm exactly in the area in which presumably the nasals reached the naris. This is supported by the fact that the remaining portion of the nasals still protrude into the naris below the missing dorsal surface. The orbits are larger than the infratemporal fenestrae; their rim is slightly upturned and their ventral edge is nearly circular. The infratemporal fenestrae are posteriorly incomplete due to the breakage of the thin quadratojugal lamina; the apparent quadratojugal spine visible on the right fenestra is interpreted here as an artifact of preservation. The supratemporal fenestrae are almond-shaped and have a smooth anteromedial corner (the fossa is not shallow in that area). The bones delimiting the fenestrae do not overhang the fossa. The medial parietal wall of the supratemporal fenestra is imperforate. The posterior margin of otic aperture is probably bowed, but the presence of matrix in the area and a possible breakage renders this statement as tentative. The foramen magnum is as broad as the occipital condyle. The incisive foramen is relatively small and abuts the premaxillary tooth row. The suborbital fenestrae extend anteriorly to the ninth alveolus. The internal choana is entirely surrounded by pterygoids and projects postero-ventrally. Its rim and internal structure is significantly altered and therefore it is not possible to evaluate the presence or absence of a septum, but the posterior area is clearly flush with the pterygoid surface and there is no evidence for a notch. Concerning the smaller foramina, the foramen of the XII cranial nerve is very small, the foramen vagi is hosted in a very wide depression, and the posterior carotid foramen is located in the depression of the foramen vagi, but is much smaller and placed below it.

### Skeletal elements

The dorsal surface of each premaxilla, close to the anterior edge, is pierced by the hole produced by the occlusion of the first dentary tooth. The surface lateral to naris is smooth. The posterior projections of the premaxillae are short: they do not reach the level of the third alveolus on the dorsal surface and just the first alveolus on the ventral surface. Each premaxilla has five alveoli. The largest alveolus is the fourth. There are 13 alveoli on the right maxilla and probably the same number on the left side. All the alveoli are circular in cross-section (slightly compressed the last ones). The maxillary tooth row is rather linear. The largest alveolus is the fifth and the penultimate alveolus has a diameter less than twice the diameter of the last one. The maxilla terminates in palatal view anterior to the lower temporal bar and its medial margin is linear and adjacent to the suborbital fenestra. The nasals are relatively broad and dorsally curved in their posterior sector (in the area hosting the rostral boss). The posterior rim of the external naris is incomplete and therefore it is not possible to directly assess the relationships between the nasals and the naris that however seem to contact each other (see the section “Fenestrae and major openings”).

The lacrimal is much longer than the prefrontal, and it makes a broad contact with nasal without any insertion of the posterior processes of maxilla. The jugal forms the posterior angle of infratemporal fenestra. The medial jugal foramen is covered by concretion, but thanks to the CT scan it is possible to state that it is small. The prefrontal dorsal surface is rather flat, without any discrete knob-like processes. Prefrontals are separated from each other by frontals and nasals. Both the prefrontal pillars are preserved, but the left one is still embedded in the matrix and does not offer any detailed morphological information. Conversely, the right pillar is clearly anteroposteriorly expanded dorsally and bears a medial process anteroposteriorly expanded and constricted at the base. The prefrontal pillar is solid without pneumatic recess. The frontal is smooth between orbits (it does not host a midsagittal crest). The frontoparietal suture is wavy and asymmetrical and fully lies on the skull table. The frontal has an acute and sharp anterior termination. The postorbital bar is slender and has a short and not prominent process. The ventral margin of postorbital bar is inset to the lateral jugal surface. The dorsal margin is not continuous with anterolateral edge of skull table, but clearly inset. The parietal is deeply constricted by the supratemporal fenestrae. The supraoccipital is rather small and it is only minimally exposed on the skull table at the back of the parietal.

The dorsal surface of the squamosals is rather flat and does not develop a marked convexity at the posterolateral edge. The squamosal does not extend ventrolaterally to lateral extent of the paroccipital process. The dorsal and ventral rims of the squamosal groove for the ear valve musculature are parallel to each other. The squamosal-quadrate suture extends dorsally along the posterior margin of the external auditory meatus. The quadrate ramus has no crest on the dorsal surface. The foramen aëreum is small, and located on the mediodorsal angle of the quadrate. The quadrate ramus has, on its ventral surface, the attachment scar for the posterior mandibular adductor muscle. The medial hemicondyle is expanded. The presence and development of the quadratojugal spine was not evaluated for preservational reasons. The putative spine visible in the right infratemporal fenestra is very likely the result of the breakage of the medial rim of the fenestra. The quadratojugal does not show an anterior process along the lower temporal bar, but extends to the superior angle of the infratemporal fenestra. The palatine process extends significantly beyond the anterior end of the suborbital fenestra and terminates by curving gently. The left palatine-pterygoid suture clearly does not reach the posterior corner of the suborbital fenestra, but the morphology is less clear on the left side. The lateral edges of palatines are parallel posteriorly. The ectopterygoid abuts maxillary tooth row. The ectopterygoid maxillary ramus forms less than two thirds of the lateral margin length of suborbital fenestra. Its anterior tip terminates at the level of the third last alveolus and is clearly forked on both sides. The left ectopterygoid extends along the medial face of the postorbital bar, and the right one extends slightly beyond. The pterygoid ramus of the ectopterygoid is bowed and the posterolateral margin of suborbital fenestra is concave. Since the pterygoid flanges are broken and missing, it is not possible to assess the ectopterygoid extension along the pterygoid. The pterygoid surface lateral and anterior to the choana is rather flush with the choanal margin. The basisphenoid is clearly thin and does not show any sulcus on the rostrum. The laterosphenoid process is oriented anteroposteriorly.

### Dentition and occlusal pattern

There are 5 and 13 alveoli for each premaxilla and maxilla (Fig. 3). Teeth are preserved in the following positions: 3, 4, 5 and 3, 4 respectively on the right and left premaxilla; 1, 2, 4, 5, 9, 10, 12 and 3, 4, 5, 9, 10, 11 respectively on the right and left maxilla. The shape of the crown varies according to its position in the tooth row. All premaxillary teeth are slender, whereas those of the dentary show a significant variation, being the posterior teeth much lower and stouter that the anterior teeth. The crowns are variably preserved, but no one is complete. However, it is clear that they are not distinctly labiolingually compressed and characterized by a mesiodistal carina, devoid of serration.

For preservational reasons (mostly the presence of the arenaceous crust in the interalveolar spaces), the only evident occlusal pit is located in the sixth interalveolar space of the right maxilla. The absence of pits medial to the tooth row in the posterior sector of the maxillae contributes in indicating that the general occlusal pattern was characterized by the dentary teeth occluding in line with the maxillary tooth row.

## Results

**Figure 4:**
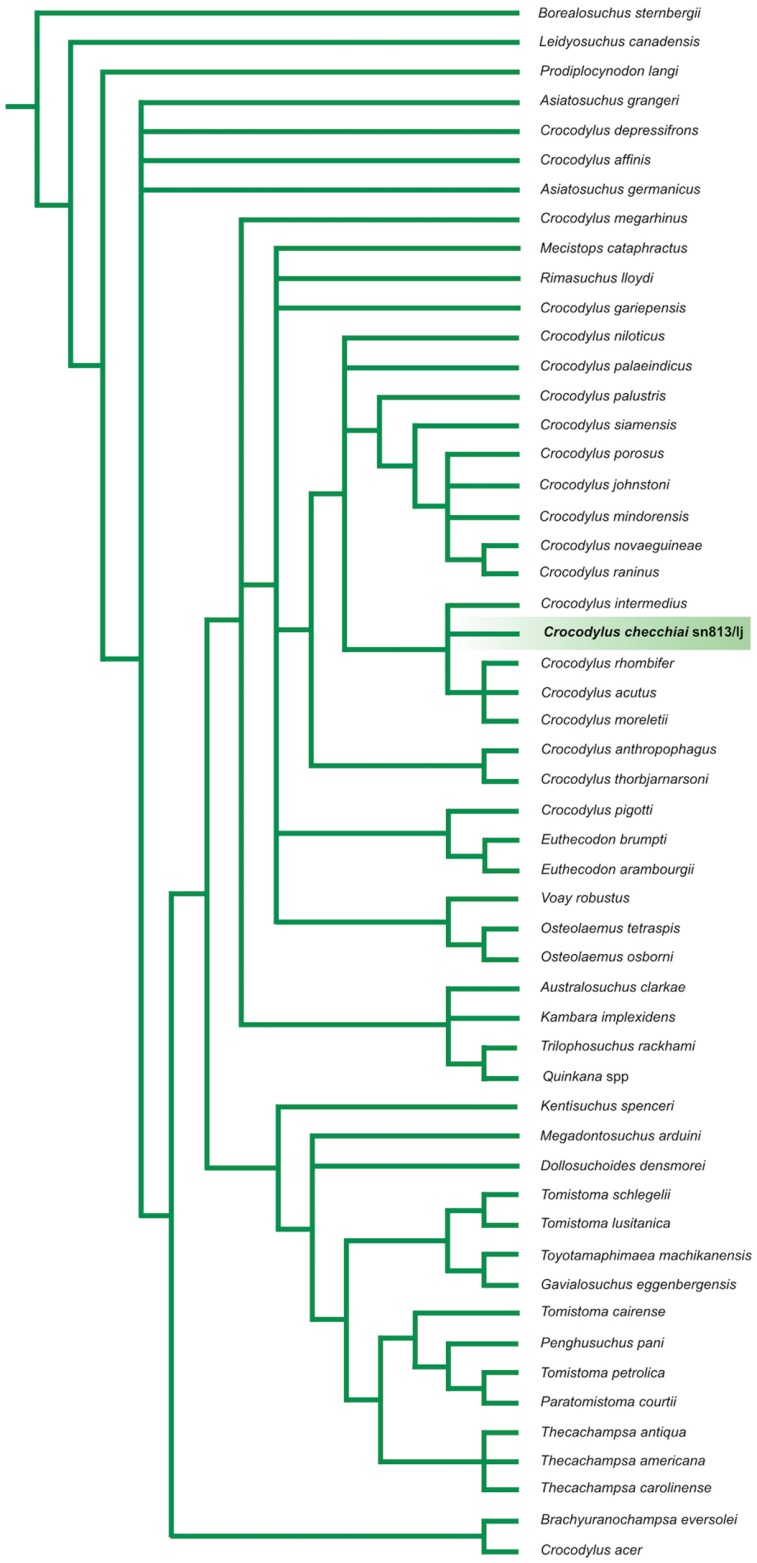
Implied weighting tree of a matrix comprising 53 taxa and 189 characters based on Brochu and Storrs^9^. *Crocodylus checchiai* sn813/lj is retrieved in a polytomy along with all the four extant American *Crocodylus*.

In the first analysis (see results in Fig. 4) we obtained 6 equally parsimonious trees 311 steps long and with adjusted homoplasy = 29.5, Consistency Index = 0.47 and Retention index = 0.71. Strict consensus tree is 327 steps long with adjusted homoplasy = 31.25, Consistency Index = 0.45 and Retention index = 0.69. *Crocodylus checchiai* is placed in a clade comprehending all South American crocodiles; it is arranged in polytomy with *C. intermedius* and basal to a polytomic clade including *C. moreletii*, *C. rhombifer* and *C. acutus*.

**Figure 5:**
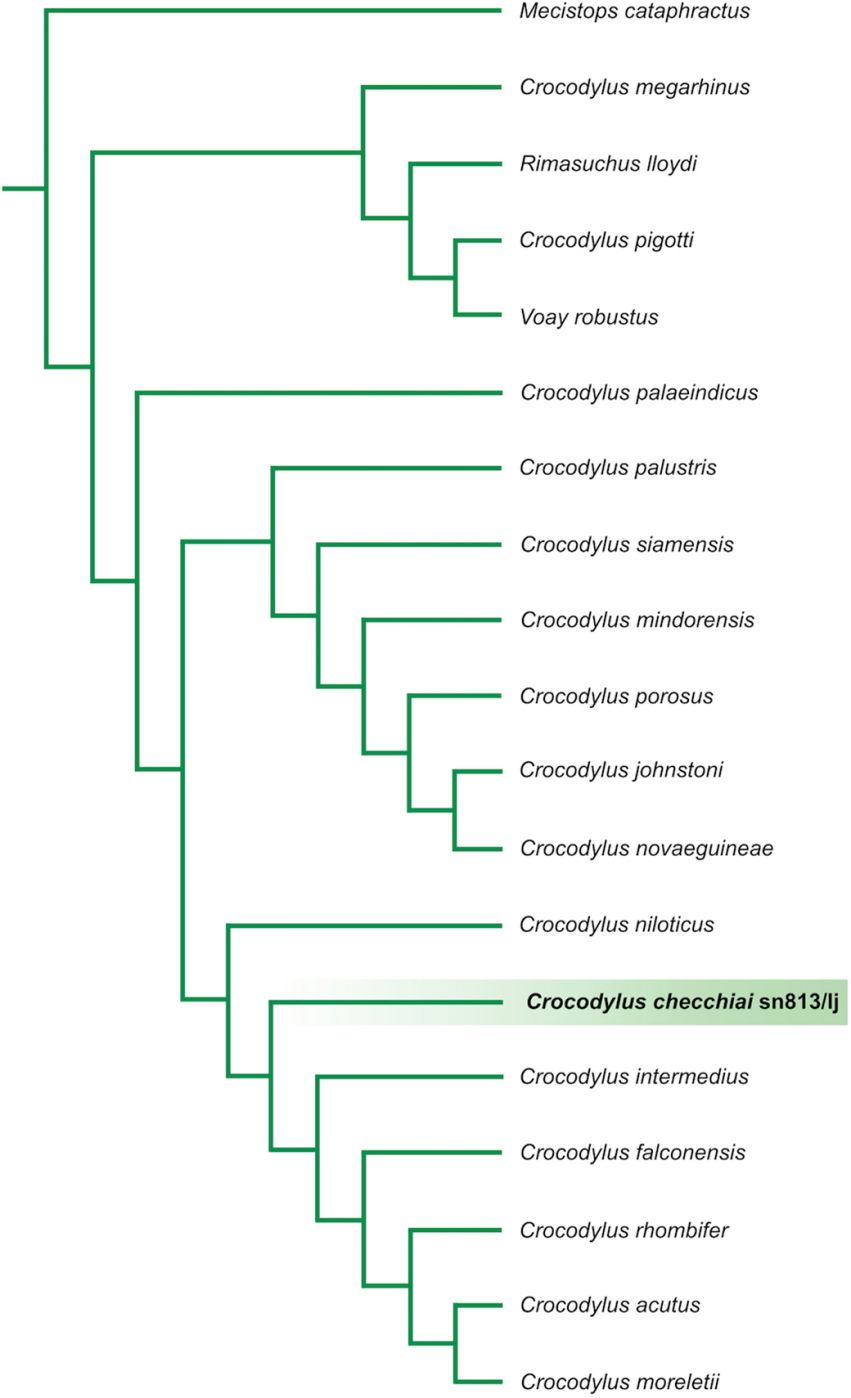
The addition of the coding of *Crocodylus checchiai* based on the late Miocene Libyan specimen sn813/lj to an updated version of the matrix by Scheyer et al.^19^ provides (“implicit enumeration search” with implied weighting) a topology with *C. checchiai* in intermediate position between the African *Crocodylus niloticus* and American crocodiles, with the early Pliocene *Crocodylus falconensis* nested within the American clade and not at its base as in Scheyer et al.^19^.

In the second analysis (see results in Fig. 5) we obtained 1 tree 64 steps long and with adjusted homoplasy = 5.99, Consistency Index = 0.55 and Retention index = 0.70. *Crocodylus niloticus* is the outgroup of the clade including, as successive ingroups, *C. checchiai*, *C. intermedius*, *C. falconensis* and all the other extant *Crocodylus* species from America.

## Discussion

### The phylogenetic relationships of *Crocodylus checchiai*

Despite the four American *Crocodylus* species share with the African extinct *Crocodylus checchiai* an evident, unique morphological character, the mid rostral boss, a close phylogenetic relationship among them was not retrieved in earlier studies.

Maccagno^14^ was aware that the median boss is present in at least some of the American crocodile species (she listed “*Cr. americanus*” and “*Cr. rombifer*” [sic]) but since she discussed this and other characters in a taxonomic context only, she simply discarded the conspecificity of the Libyan taxon without facing its phylogenetic relationships. On the contrary, on the basis of a mixture of characters of little or no phylogenetic value, and not taking into consideration intra and interspecific variability and the effects of postmortem deformation, Maccagno^15^ identified in the extinct *Crocodylus palaeindicus* and the Asian taxa the closest relationships of the Libyan crocodylians. As already remarked by Brochu^6^, Tchernov^24^ considered *C. checchiai* as the sister taxon of *C. niloticus*, whereas according to Leakey et al.^25^ these two taxa were even synonyms. Hect^13^ correctly underlined a link with the American taxa by stating that “the similarity of the dorsal skull morphology of *Crocodylus checchiai* to the two forms of American freshwater crocodilians, *Crocodylus moreletti* [sic] and *C. rhombifera* [sic], indicated that these two forms may be the relics of a more widespread species distribution”. He concluded that even if it could be a homoplastic character, the promontorium should be considered a synapomorphy of the group on the basis of the knowledge of that time.

The first, and up to now only, phylogenetic analysis that evaluated the relationships of *C. checchiai*^9,26^ confirmed the inclusion of this species in the genus *Crocodylus*, but, having retrieved a broad politomy, lacks of enough resolution to provide precise indications.

**Figure 6:**
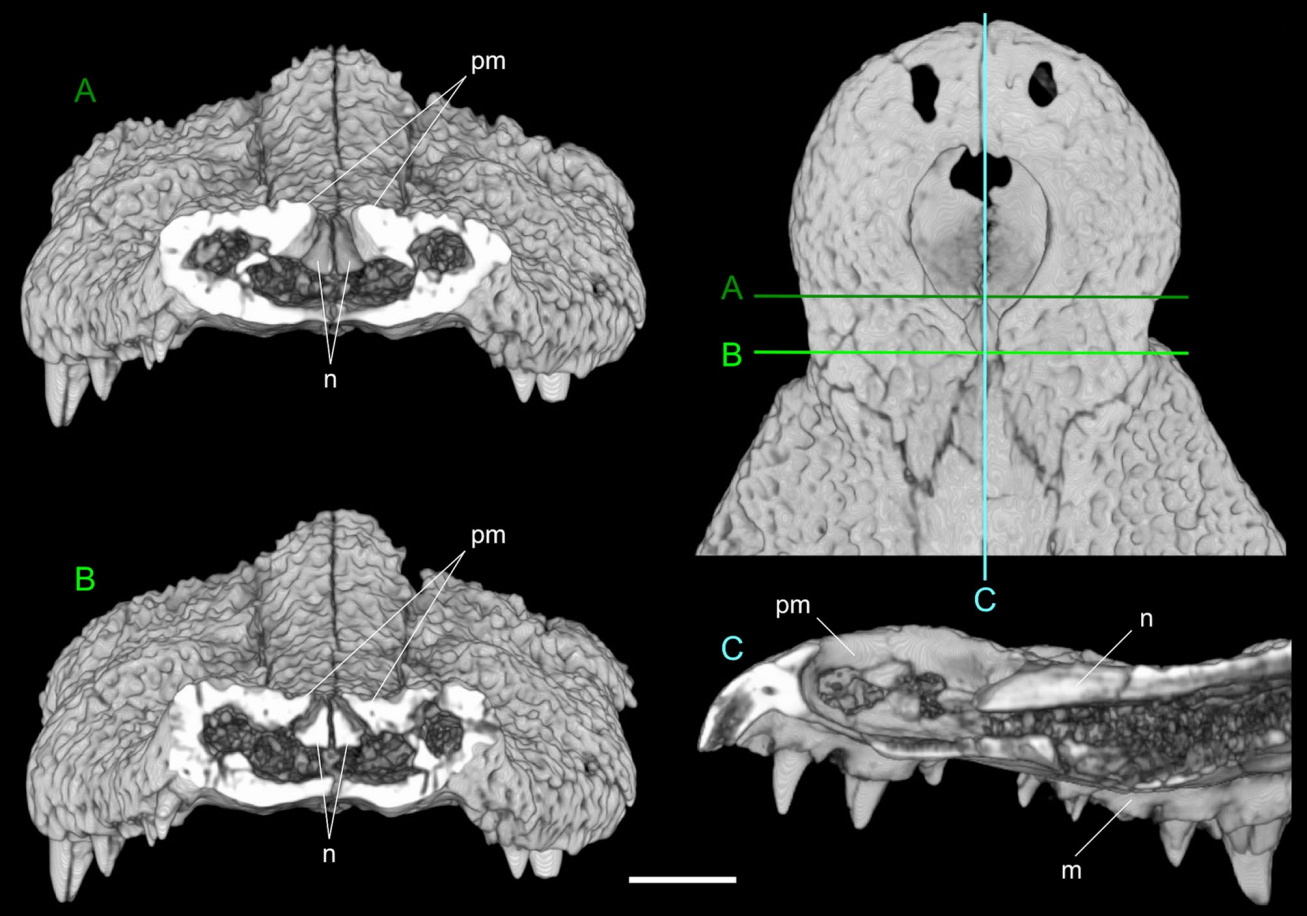
CT investigation of the relationship between the nasals and the premaxillae in sn813/lj. Top left: anterior tip of the snout in dorsal view showing the planes of the sections in (**A**), (**B**) and (**C**). (**A**) anterior transversal section do not involve the nasals; (**B**): posterior transversal section cutting the nasals and showing that the premaxillae are entirely separated by the nasals; (**C**): sagittal section showing that the nasals are slightly lowered in the area of the posterior process of the premaxillae. Note in (**A**) and (**B**) how the nasals are raised to form the medial boss of the snout. Scale bar: 3 cm.

The scoring of *C. checchiai* by Brochu and Storrs^9^ was based on the two skulls KNM-LT 23108 and KNM-LT 26618 as well as postcranial material from the late Miocene-early Pliocene of Tanzania and differs from the scoring we used (based on sn813/lj) for a few characters (for further discussion see Supplementary Note 1). In particular, two characters were scored differently in the two matrixes (and 29 characters were scored in Brochu and Storrs^9^, but not by us; ten characters were scored by us for the first time). The anterior process of the ectoperygoids is cleary forked in sn813/lj but was scored as pointed in Brochu and Storrs^9^. Remarkable is the relationship between the nasals and the external naris because on one side Maccagno clearly stated that in both the now lost holotype of *C. checchiai* and sn813/lj (as we confirmed; see Fig. 6) the nasals participated in the posterior rim of the external naris, as described and shown by Delfino^18^ for specimen 30P24A likewise coming from As Sahabi (further inspection of the latter confirmed that the nasals separate the premaxillae behind the naris for about 10 mm). On the other side, Hect^13^ described and figured the opposite condition in 3P84A from the same area. The fact that the material from Tanzania shows nasals not reaching (at least externally) the naris should not by itself grant specific separation, but requires further evaluation of the possible polymorphy of the character. The CT scans did not allow us to confirm the presence of the linear array of pits in the caviconchal recesses in sn813/lj, whose presence was considered as unknown in *C. checchiai*^9^ even if character 101 was scored as 1 (presence of a linear array of pits) in their matrix.

### Towards the origin of the Neotropical crocodiles

According to the results obtained by Oaks^2^, there are two possible scenarios for the dispersal of *Crocodylus* to the Neotropics: from Australasia to the Neotropics and then to Africa or, alternatively, from Australasia to Africa and then to the Neotropics.

Thanks to our results, the late Miocene *Crocodylus checchiai* could offer a geographic and phylogenetic link between the African *Crocodylus niloticus* and the American clade whose oldest member is *C. falconensis* from the early Pliocene, providing direct evidence for the dispersal from Africa to America and suggesting to discard the other option. As shown in Fig. 4, resulting from the analysis based on the data matrix by Brochu and Storrs^9^, but with the scoring of the African *C. checchiai* sn813/lj from Libya, the latter belongs to the American clade, even if the resolution of the retrieved trees does allow us to place it, as theoretically expected because of its age, at the base of the extant Neotropical taxa.

Conversely, the application of the implied weighting method to an updated version of the smaller matrix (only 32 characters and 18 taxa) published by Scheyer et al.^19^, with the addition of our scoring of *C. checchiai*, provides a well-resolved topology (Fig. 5) that places *C. niloticus* at the base of a branch including as successive ingroups *C. checchiai*, *Crocodylus intermedius*, *Crocodylus falconensis*, and then the other extant American species (for further discussion see Supplementary Note 2). The extinct *C. falconensis* was recently described from the early Pliocene of Venezuela^19^ and, representing the geologically oldest palaeontological evidence of the presence of *Crocodylus* in America, provides a term ante quem for the dispersal of *Crocodylus*. As for the fossil record of *Crocodylus* in America, to the remains mentioned by Brochu^6^ we should add the recently described fragmentary remains from the late Pliocene Ware Formation (Colombia) referred to *Crocodylus* sp. by Moreno-Bernal et al.^27^. None of them is older than the early Pliocene and, in fact, Nicolai and Mazke^3^ considered as Pliocene the dispersal of *Crocodylus* from Africa to America.

The late Miocene age of the African *Crocodylus checchiai* slightly predates previous estimates and is however fully congruent with a westward dispersal towards America and the appearance of *Crocodylus* in America in the early Pliocene fossil record. It seems likely that at the same time *Crocodylus* dispersed northward from Africa to Europe across the Tethys (or alternatively from Asia to Europe^3^) as testified by several remains coming from different late Miocene southern European localities. At least the remains from the fissures fillings of Gargano promontory^28^ can be confidently referred to *Crocodylus* sp., but also the remains from Montebamboli^29^ and Scontrone^30^ have been tentatively referred to the same taxon. Interestingly enough, the presence of *C. checchiai* has been cited for the Iberian late Miocene site of Venta del Moro^31^, but a recent revision of this fragmentary material did not confirm the specific identification and suggested that the material could belong to an undetermined crocodile (Delfino, personal observations).

Remarkably, thanks to the evolution of range-expansion-promoting traits (salt tolerance^32^, but not only, see Nicolai and Mazke^3^) *Crocodylus* started to disperse across the globe very quickly around the end of the Miocene and successfully replaced the local taxa that went extinct because of climatic changes and related environmental remodeling^6,19,27,30,33,34,35^.

## Methods

### Tomographic analysis

Given the rarity of complete and well preserved skulls of *C. checchiai*, the non-destructive method of CT scanning^36–39^ was chosen to observe and analyse the internal anatomical features of the specimen sn813/lj. The fossil was scanned in its entirety in the coronal slice plane from front to back using a Philips Brilliance CT 64-channel scanner at M.G. Vannini Hospital (Rome). The scanning resulted in 670 slices with dimensions of 531 × 531 pixels. The slice thickness is 0.8 mm with an interslice space of 0.4 mm. The CT data were processed using OsiriX 5.5.2 and Materialise Mimics 20.0. The final 3D model was rendered with ZBrush 4R6.

### Phylogenetic analysis

We performed two different phylogenetic analyses in TNT software^40^ (Supplementary Data Files S1-S2). The first analysis was based on Brochu and Storrs^9^: we replaced their coding of *Crocodylus checchiai* based on Tanzanian material with our coding of the same taxon based on the Libyan specimen sn813/lj (Supplementary Data Files S1 and Supplementary Note 3; for an alternative analysis with both Libyan and Tanzanian codings see Supplementary Note 4; Supplementary Fig. S1 and Supplementary Data File S3), thus building a matrix comprising 53 taxa and 189 characters. *Borealosuchus sternbergii* was set as outgroup. We performed a “new technology search”^41^. Characters were weighted equally, and multistate characters were left unordered.

In the second analysis, we added our coding of *Crocodylus checchiai* (Supplementary Note 5) to an updated version of the matrix published in Scheyer et al.^19^ that includes also *C. falconensis*, i.e. an extinct species that represent the oldest *Crocodylus* from South America. The matrix comprised therefore 19 taxa and 32 characters (Supplementary Data File S2). In this case we performed an “implied weighting” with ùk parameter = 3^41^. Multistate characters were left unordered.

## Supplementary information

Supplementary Information 1.

Supplementary Information 2.

Supplementary Information 3.

Supplementary Information 4.

Supplementary Information 5.
