# Supplementary material for: Old African fossils provide new evidence for the origin of the American crocodiles
Source: Sci Rep. 2020 Jul 23;10:11127. doi: 10.1038/s41598-020-68482-5 (PMC7378212; doi:10.1038/s41598-020-68482-5)
Supplement: Supplementary file 1 — Supplementary Information 1. [file 41598_2020_68482_MOESM1_ESM.pdf]

Supplementary Data File

Old African fossils provide new evidence for the origin of the American crocodiles

Massimo Delfino, Dawid A. Iurino, Bruno Mercurio, Paolo Piras, Lorenzo Rook & Raffaele Sardella

Matrix Brochu & Storrs with *C. checchiai* Libya only

Borealosuchus sternbergii				0	0	0	0	0	0	0	0	0	?
	1	1	0	0	1	0	?	1	0	0	1	0	0
	0	0	0	0	1	0	1	0	0	0	0	0	1
	?	0	0	?	?	?	0	?	?	?	0	1	1
	0	2	0	0	0	0	0	0	0	?	0	0	0
	2	0	0	0	0	0	0	0	1	0	0	0	0
	0	?	?	0	0	0	2	0	?	0	0	0	0
	0	0	0	1	3	1	0	0	0	1	0	0	0
	1	0	0	0	0	0	1	?	0	?	0	0	0
	0	0	1	1	1	0	1	0	0	0	1	0	1
	0	0	0	0	0	1	1	1	1	0	0	?	0
	0	0	1	0	0	?	0	0	0	0	0	0	1
	0	0	1	0	1	0	0	0	0	0	0	?	1
	?	0	0	1	0	0	1	1	0	0	0	0	0
	0	0	?	?	?	0	0	0	0	1			

Leidyosuchus canadensis				?	?	?	?	0	?	0	?	?	?
	?	?	?	?	1	?	?	?	?	0	1	0	0
	0	0	0	1	1	?	1	?	?	1	0	?	0
	?	1	1	?	?	1	1	?	?	?	0	1	1
	0	?	0	0	0	0	0	?	0	?	0	1	?
	2	0	0	0	0	0	0	1	1	1	0	1	?
	?	?	?	0	0	0	1	0	?	0	0	0	0
	0	0	0	0	3	0	0	0	0	1	0	0	0
	0	0	1	0	0	0	1	0	0	0	1	0	0
	0	0	1	1	1	0	1	0	0	0	1	0	1
	0	0	0	1	0	1	1	1	1	0	0	?	0

1 0 1 0 0 1 0 0 1 0 0 0 1
0 0 1 0 1 0 0 0 0 0 0 0 1
? 0 0 1 0 0 1 1 0 0 1 0 0
0 1 ? ? ? 0 0 0 0 1

Mecistops cataphractus 1 0 ? 0 0 1 0 0 1 ?
0 0 0 0 1 0 0 0 0 0 1 1 1
0 0 1 1 1 1 1 2 0 1 2 0 0
0 1 1 1 1 0 1 1 0 1 1 1 1
0 4 1 0 1 0 1 0 0 0 1 0 0
2 0 0 0 1 1 1 0 1 0 1 ? 1
0 0 1 0 0 1 2 0 0 0 0 0 0
1 0 0 2 1 0 0 0 0 1 0 0 0
1 0 0 0 0 0 1 1 0 1 0 0 0
0 1 0 1 1 0 1 0 0 0 1 0 1
0 0 0 0 0 1 1 1 1 1 0 0 0
0 1 0 1 1 1 0 0 1 2 0 0 1
0 0 1 0 1 0 0 0 1 1 1 0 1
0 1 1 1 1 0 0 1 0 0 0 0 0
0 3 0 0 ? 1 1 1 0 1

Crocodylus niloticus 1 0 1 0 0 0 0 0 1 ? 1
0 1 0 1 0 0 0 1 0 1 1 1 0
0 1 1 1 1 1 2 0 1 2 0 0 0
1 1 1 2 0 1 1 0 1 1 1 0 0
2 1 0 1 0 1 0 0 0 1 0 1 2
0 0 0 1 1 1 0 1 0 1 1 1 0
0 1 0 0 1 1 0 0 0 0 0 0 1
0 0 2 1 0 0 0 0 1 0 0 1 1
0 0 0 0 0/1 1 1 0 1 0 0 0/1 0
0 0 1 1 0 1 0 0 0 1 0 1 1
0 0 0 0 1 1 1 1 1 0 0 0 0
1 0 1 1 1 0 0 1 2 0 0 1 0
0 1 0 1 0 0 0 1 1 1 0 1 0
1 1 1 1 0 0 1 1 0 0 0 0 0
3 0 1 1 0 0 0 0 1

Crocodylus porosus 1 1 1 0 0 0 0 0 1 ? 0
0 1 0 1 0 1 0 1 0 1 1 1 0
0 0 1 1 1 1 2 0 1 2 0 0 0
1 1 1 2 0 1 1 0 1 1 1 0 0
2 1 0 1 0 1 0 0 0 1 0 1 2
0 0 0 1 1 1 0 1 0 1 1 1 0
0 1 0 0 1 1 0 0 0 0 0 0 1
0 0 2 1 0 0 0 1 1 0 0 1 1
0 0 0 0 0 1 1 0 1 0 0 0/1 0
0 0 1 1 0 1 0 0 0 1 0 1 0

0	0	0	0	1	1	1	1	1	0	0	0	0
1	0	1	1	1	0	0	1	2	0	0	1	0
0	1	0	1	0	0	0	1	1	1	0	1	0
1	1	1	1	0	0	1	1	0	0	0	0	0
3	0	1	1	0	0	0	0	1				
Crocodylus rhombifer		0	0	1	0	0	0	0	0	1	?	1
0	1	0	1	0	0	0	1	0	1	1	1	0
0	1	1	1	1	1	2	0	1	1	0	0	0
1	1	1	2	0	1	1	0	1	1	1	0	0
2	1	0	1	0	1	0	0	0	1	0	1	2
0	0	0	1	1	1	0	1	0	1	1	1	0
0	1	0	0	1	1	0	0	0	0	0	0	1
0	0	2	1	0	1	0	0	1	0	0	1	1
0	0	0	0	0	1	1	0	1	0	0	0/1	0
0	0	1	1	0	1	0	0	0	1	0	1	1
0	0	0	0	1	1	1	1	1	0	0	0	0
1	0	1	1	1	0	0	1	2	0	0	1	0
0	1	1	1	0	0	0	1	1	1	0	1	0
1	1	1	1	0	0	1	1	0	0	0	0	0
3	0	1	2	0	0	0	0	1				
Crocodylus acutus		0	0	1	0	0	0	0	0	1	?	1
0	1	0	1	1	0	0	1	0	1	1	1	0
0	1	1	1	1	1	2	0	1	2	0	0	0
1	1	0	2	0	1	1	0	1	1	1	0	0
2	1	0	1	0	1	0	0	0	1	0	1	2
0	0	0	1	1	1	0	1	0	1	1	1	0
0	1	0	0	1	1	0	0	0	0	0	0	1
0	0	2	1	0	1	0	0	1	0	0	1	1
0	0	0	0	0	1	1	0	1	0	0	0/1	0
0	0	1	1	0	1	0	0	0	1	0	1	1
0	0	0	0	1	1	1	1	1	0	0	0	0
1	0	1	1	1	0	0	1	2	0	0	1	0
0	1	0	1	0	0	0	1	1	1	0	1	0
1	1	1	1	0	0	1	1	0	0	0	0	0
3	0	1	2	0	0	0	0	1				
Crocodylus palustris		1	0	1	0	0	0	0	0	1	?	0
0	1	0	1	0	0	1	1	0	1	1	1	0
0	1	1	1	1	1	2	1	1	2	1	0	0
1	1	1	2	0	1	1	0	1	1	1	0	0
2	1	0	1	0	1	0	0	0	1	0	1	2
0	0	0	1	1	1	0	1	0	1	1	1	0
0	1	0	0	1	1	0	0	0	0	0	0	1
0	0	2	1	0	0	0	1	1	0	0	1	1
0	0	0	0	0	1	1	0	1	0	0	0/1	0

0	0	1	1	0	1	0	0	0	1	0	1	0
0	0	0	0	1	1	1	1	1	0	0	0	0
1	0	1	1	1	0	0	1	2	0	0	1	0
0	1	0	1	0	0	0	1	1	1	0	1	0
1	1	1	1	0	0	1	1	0	0	0	0	0
3	0	1	1	0	0	0	0	1				
Crocodylus siamensis		1	1	1	0	0	0	0	0	1	?	1
1	1	0	1	0	0	0	0	0	1	1	1	0
0	1	0	1	1	1	2	0	1	2	0	0	0
1	1	1	2	0	1	1	0	1	1	1	0	0
2	1	0	1	0	1	0	0	0	1	0	1	2
0	0	0	1	1	1	0	1	0	1	1	1	0
0	1	0	0	1	1	0	0	0	0	0	0	1
0	0	2	1	0	0	0	1	1	0	0	1	1
0	0	0	0	0	1	1	0	1	0	0	0/1	0
0	0	1	1	0	1	0	0	0	1	0	1	0
0	0	0	0	1	1	1	1	1	0	0	0	0
1	0	1	1	1	0	0	1	2	0	0	1	0
0	1	1	1	0	0	0	1	1	1	0	1	0
1	1	1	1	0	0	1	1	0	0	0	0	0
3	0	1	1	0	0	0	1	1				
Crocodylus intermedius		0	0	1	0	0	0	0	0	1	?	1
0	1	0	1	0	0	0	1	0	1	1	1	0
0	1	1	1	1	1	2	0	1	2	0	0	0
1	1	1	2	0	1	1	0	1	1	1	1	0
2	1	0	1	0	1	0	0	0	1	0	1	2
0	0	0	1	1	1	0	1	0	1	1	1	0
0	1	0	0	1	1	0	0	0	0	0	0	1
0	0	2	1	0	1	0	0	1	0	0	1	1
0	0	0	0	0	1	1	0	1	0	0	0/1	0
0	0	1	1	0	1	0	0	0	1	0	1	0
0	0	0	0	1	1	1	1	1	0	0	0	0
1	0	1	1	1	0	0	1	2	0	0	1	0
0	1	0	1	0	0	0	1	1	1	0	1	0
1	1	1	1	0	0	1	1	0	0	0	0	0
3	0	1	2	0	0	0	0	1				
Crocodylus johnstoni		1	1	1	0	0	0	0	0	1	?	0
0	1	0	1	0	1	0	1	0	1	1	1	0
0	0	1	1	1	1	2	0	1	2	0	0	0
1	1	1	2	0	1	1	0	1	1	1	?	0
2	1	0	1	0	1	0	0	0	1	0	1	2
0	0	0	1	1	1	0	1	0	1	1	1	0
0	1	0	0	1	1	0	0	0	0	0	0	1
0	0	2	1	0	0	0	1	1	0	0	1	1

0	0	0	0	0	1	1	0	1	0	0	0/1	0
1	0	1	1	0	1	0	0	0	1	0	1	0
0	0	0	0	1	1	1	1	1	0	0	0	0
1	0	1	1	1	0	0	1	2	0	0	1	0
0	1	0	1	0	0	0	1	1	1	0	1	0
1	1	1	1	0	0	1	1	0	0	0	0	0
3	0	1	1	0	0	0	0	1				
Crocodylus mindorensis			1	1	1	0	0	0	0	0	1	?
0	0	1	0	1	0	1	0	1	0	1	1	1
0	0	0	1	1	1	1	2	0	1	2	0	0
0	1	1	1	2	0	1	1	0	1	1	1	0
0	2	1	0	1	0	1	0	0	0	1	0	1
2	0	0	0	1	1	1	0	1	0	1	1	1
0	0	1	0	0	1	1	0	0	0	0	0	0
1	0	0	2	1	0	0	0	1	1	0	0	1
1	0	0	0	0	0	1	1	0	1	0	0	0/1
0	0	0	1	1	0	1	0	0	0	1	0	1
0	0	0	0	0	1	1	1	1	1	0	0	0
0	1	0	1	1	1	0	0	1	2	0	0	1
0	0	1	0	1	0	0	0	1	1	1	0	1
0	1	1	1	1	0	0	1	1	0	0	0	0
0	3	0	1	1	0	0	0	0	1			
Crocodylus novaeguineae			1	1	1	0	0	0	0	0	1	?
0	0	1	0	1	0	1	0	1	0	1	1	1
0	0	0	1	1	1	1	2	0	1	2	0	0
0	1	1	1	2	0	1	1	0	1	1	1	0
0	2	1	0	1	0	1	0	0	0	1	0	1
2	0	0	0	1	1	1	0	1	0	1	1	1
0	0	1	0	0	1	1	0	0	0	0	0	0
1	0	0	2	1	0	0	0	1	1	0	0	1
1	0	0	0	0	0	1	1	0	1	0	0	0/1
0	0	0	1	1	0	1	0	0	0	1	0	1
0	0	0	0	0	1	1	1	1	1	0	0	0
0	1	0	1	0	1	0	0	1	2	0	0	1
0	0	1	0	1	0	0	0	1	1	0	0	1
0	1	1	1	1	0	0	1	1	0	0	0	0
0	3	0	1	1	0	0	0	0	1			
Crocodylus raninus		?	?	?	?	?	?	?	?	?	?	?
?	?	?	?	?	?	?	?	?	?	?	?	?
?	?	?	?	?	?	?	?	?	?	?	?	?
?	?	?	?	?	?	?	?	?	?	?	?	?
?	?	?	?	?	?	?	?	?	?	?	?	?
?	?	?	?	?	?	?	?	?	?	?	?	?
?	?	0	0	1	1	0	?	0	0	0	0	1

0	0	2	1	0	0	0	1	1	0	0	1	1
0	0	0	0	0	1	1	0	1	0	0	1	0
0	0	1	1	0	1	0	0	0	1	0	1	0
0	0	0	0	1	1	1	1	1	0	0	0	0
1	0	1	0	1	0	0	1	2	0	0	1	0
0	1	0	1	0	0	0	1	1	0	0	1	0
1	1	0	1	0	0	1	1	0	3	0	0	0
0	?	?	?	0	0	0	0	?				
Crocodylus moreletii		0	0	1	0	0	0	0	0	1	?	1
0	1	0	1	0	0	0	1	0	1	1	1	0
0	1	1	1	1	1	2	0	1	2	0	0	0
1	1	1	2	0	1	1	0	1	1	1	0	0
2	1	0	1	0	1	0	0	0	1	0	1	2
0	0	0	1	1	1	0	1	0	1	1	1	0
0	1	0	0	1	1	0	0	0	0	0	0	1
0	0	2	1	0	1	0	0	1	0	0	1	1
0	0	0	0	0	1	1	0	1	0	0	0/1	0
0	0	1	1	0	1	0	0	0	1	0	1	1
0	0	0	0	1	1	1	1	1	0	0	0	0
1	0	1	1	1	0	0	1	2	0	0	1	0
0	1	0	1	0	0	0	1	1	1	0	1	0
1	1	1	1	0	0	1	1	0	0	0	0	0
3	0	1	2	0	0	0	0	1				
Crocodylus palaeindicus			?	?	?	?	?	?	0	?	?	?
?	?	?	?	?	?	?	?	?	0	?	1	?
?	?	?	?	?	?	?	?	?	1	?	?	?
?	?	?	?	?	?	?	?	?	?	1	1	0
0	2	1	0	1	0	1	?	0	0	1	0	1
2	0	0	0	1	1	?	0	1	?	1	1	?
?	?	?	0	0	1	1	0	?	0	0	0	0
?	0	0	2	1	0	0	0	0	1	0	0	?
1	0	0	0	0	0	1	1	0	1	0	0	1
0	0	0	0	1	0	1	0	0	?	1	0	1
0	0	0	0	0	1	1	1	1	1	0	?	0
0	1	0	1	1	?	0	0	1	2	0	0	1
0	0	1	0	1	0	1	0	0	1	1	?	1
?	1	1	1	1	0	0	1	1	0	0	0	0
0	3	?	?	?	0	0	0	?	1			
Crocodylus anthropophagus			?	?	?	?	?	?	?	?	?	?
?	?	?	?	1	0	?	?	?	0	1	?	1
0	0	1	1	1	?	?	?	?	1	2	0	0
?	1	1	?	?	?	1	?	?	?	1	1	?
0	2	1	?	1	?	1	?	0	?	1	0	?
2	0	?	0	1	1	1	0	1	0	1	1	?

?	?	?	0	0	0	1	0	?	0	0	0	0
1	0	0	2	1	0	0	0	0	1	0	?	1
1	0	0	0	0	1	1	1	0	1	0	?	0
?	?	?	1	?	0	1	0	0	?	?	0	1
?	0	0	0	?	1	1	1	1	1	0	?	?
?	1	?	1	0	?	0	0	1	1	0	0	1
0	0	1	1	1	0	0	?	1	?	1	?	1
?	?	1	1	1	0	0	1	1	0	0	0	0
0	3	?	?	?	?	?	?	0	?			
Crocodylus thorbjarnarsoni			?	?	?	?	?	?	?	?	?	?
?	?	?	?	?	?	?	?	?	?	?	?	?
?	?	?	?	?	?	?	?	?	?	?	?	?
?	?	?	?	?	?	?	?	?	?	1	1	?
0	2	1	0	1	0	1	0	0	0	1	0	1
2	0	0	0	1	1	1	0	1	0	1	1	?
?	?	?	0	0	0	1	0	?	0	0	0	0
1	0	0	2	1	0	0	0	0	1	0	0	?
?	0	0	0	0	1	1	?	?	?	0	0	0
0	0	0	1	1	0	1	0	0	0	0	0	1
?	0	0	0	0	1	1	1	1	1	0	?	0
0	1	?	1	0	1	0	0	1	2	0	0	1
0	0	1	1	1	0	?	?	1	1	1	?	1
?	?	?	1	1	0	0	1	1	0	0	0	0
0	3	?	?	?	0	0	0	0	?			
Euthecodon brumpti		?	?	?	?	?	?	?	?	?	?	?
?	?	?	?	?	?	?	?	?	?	?	?	?
?	?	?	?	?	?	?	?	?	?	?	?	?
1	0	?	?	?	1	?	?	?	1	1	2	2
4	?	0	4	?	?	?	?	?	1	0	0	2
0	?	0	1	1	1	0	1	0	1	0	?	?
?	?	0	0	0	2	0	?	0	0	?	0	1
1	0	2	5	0	0	0	1	1	0	0	0	1
0	0	0	0	0	?	?	?	?	0	0	1	0
0	0	1	1	0	1	0	1	0	0	0	1	0
0	0	0	0	1	1	1	1	1	0	?	?	?
1	0	1	1	1	0	0	1	1	1	0	1	0
0	1	1	1	0	0	?	1	1	1	?	1	?
?	1	1	1	0	0	1	0	0	0	0	0	0
3	?	?	?	0	0	0	0	?				
Euthecodon arambourgii			?	?	?	?	?	?	?	?	?	?
?	?	?	?	?	?	?	?	?	?	?	?	?
?	?	?	?	?	?	?	?	?	?	?	?	?
?	?	?	?	?	?	?	?	?	?	?	?	?
?	?	?	?	0	?	?	?	?	?	?	?	?

?	?	?	?	?	?	?	?	?	?	1	?	?
?	?	?	0	0	0	2	0	?	0	0	0	0
?	1	0	2	5	0	0	0	1	1	0	0	?
1	0	0	0	0	0	1	1	0	1	0	0	0
0	0	0	?	?	0	?	?	?	?	0	?	?
?	0	0	0	0	1	1	1	1	1	0	?	?
1	1	0	1	1	?	0	0	1	2	1	0	1
0	0	1	0	1	1	0	0	?	?	?	?	1
?	?	1	1	?	0	?	1	0	0	0	?	0
0	3	?	?	?	0	0	0	0	?			
Osteolaemus tetraspis		?	?	1	?	0	0	0	0	1	?	0
0	1	0	1	0	1	0	0	0	1	1	1	0
0	1	1	1	1	1	2	0	1	1	1	0	0
1	1	1	1	1	1	1	0	1	1	1	0	0
2	1	0	1	0	1	0	0	0	1	0	1	2
0	0	0	0	1	1	0	1	0	1	1	1	0
0	1	1	0	1	0	0	0	0	0	1	0	1
0	0	2	1	0	0	0	1	1	0	0	0	1
0	0	0	0	0	1	1	0	1	1	0	0	1
0	1	1	1	0	1	0	1	0	1	0	1	0
0	0	1	0	1	1	1	1	1	0	1	0	0
1	0	1	0	1	0	0	1	2	1	1	1	0
0	1	0	1	1	0	0	1	1	1	0	1	0
1	1	1	1	0	1	1	0	0	0	0	0	0
3	1	1	0	0	0	0	0	1				
Osteolaemus osborni		?	?	1	?	0	0	0	0	1	?	0
0	1	0	1	0	1	0	0	0	1	1	1	0
0	1	1	1	1	1	2	0	1	1	1	0	0
1	1	1	1	1	1	1	0	1	1	1	0	0
2	1	0	1	0	1	0	0	0	0	0	1	2
0	0	0	0	1	1	0	1	0	1	1	1	0
0	1	1	0	1	1	0	0	0	0	1	0	1
0	0	2	1	0	0	0	1	1	0	0	0	1
0	0	0	0	0	1	1	0	1	1	0	0	1
0	0	1	0	0	1	0	1	0	1	0	1	0
0	0	0	0	1	1	1	1	1	0	1	0	0
1	0	1	0	1	0	0	1	2	1	1	1	0
0	1	0	1	1	0	0	1	1	1	0	1	0
1	1	1	1	0	1	1	0	0	0	0	0	0
3	1	0	0	0	0	0	0	1				
Voay robustus	?	?	?	?	?	?	0	?	?	?	?	?
?	?	?	0	?	?	?	0	1	1	?	?	?
?	?	1	1	1	?	?	1	1	1	0	?	?
?	?	?	?	1	?	?	?	1	1	1	0	2

1	0	1	0	1	0	0	0	0	0	1	2	0
0	0	1	1	1	1	1	0	1	1	?	?	?
?	0	0	1	1	0	?	0	0	0	0	?	0
0	2	1	0	0	0	1	1	0	0	0	1	0
0	0	0	0	1	1	0	1	0	0	0	1	0
1	1	1	0	1	0	1	0	1	0	1	0	0
0	0	0	1	1	1	1	1	0	?	0	0	1
0	1	1	1	0	0	1	2	0	1	1	0	0
1	1	1	1	1	0	?	1	1	0	1	?	1
1	1	1	0	0	1	0	0	0	0	0	0	3
?	?	?	0	0	0	0	1					
Rimasuchus lloydi		?	?	?	?	?	?	?	?	?	?	?
?	?	?	?	?	?	?	?	?	?	?	?	?
?	?	?	?	?	?	?	?	?	?	?	?	?
?	?	?	?	?	?	?	?	?	?	?	?	?
?	?	?	1	?	?	?	?	?	?	?	?	?
?	?	?	?	?	?	?	?	?	1	?	?	?
?	?	0	0	1	1	0	?	0	0	0	0	?
0	0	2	1	0	0	0	1	1	0	0	?	1
0	0	0	0	0	1	1	0	1	0	0	0	0
0	?	?	?	?	1	0	?	?	1	?	?	1
0	0	0	?	1	1	1	1	1	0	?	0	0
1	0	1	1	?	0	0	1	2	0	0	1	0
0	1	0	1	1	0	0	1	1	1	?	1	?
?	1	1	1	0	?	1	0	0	0	?	0	0
3	?	?	?	?	?	0	0	?				
Crocodylus pigotti		?	?	?	?	?	?	?	?	?	?	0
0	?	0	1	0	?	0	0	?	1	1	?	?
0	1	1	1	1	?	?	?	?	?	?	?	?
1	0	?	?	?	0	?	?	?	1	1	1	0
2	?	?	1	?	?	?	?	?	?	?	?	2
?	?	?	1	1	1	?	?	?	1	?	?	?
?	?	0	0	0	1	0	?	0	1	0	0	?
0	0	2	1	0	0	0	1	1	0	0	0	1
0	0	0	0	0	?	?	?	?	0	0	1	0
1	0	1	1	0	1	0	1	0	0	0	1	?
0	?	0	0	1	1	1	1	1	0	?	?	?
1	?	1	?	?	0	0	1	2	1	0	1	0
0	1	0	1	1	0	0	?	?	1	?	1	?
?	1	1	0	0	?	1	0	0	0	0	0	0
3	?	?	?	0	0	0	0	?				
Crocodylus gariepensis		?	?	?	?	?	?	?	?	?	?	?
?	?	?	?	?	?	?	?	0	1	1	?	?
0	1	1	1	?	?	?	?	?	?	0	0	?

1	0	?	?	?	1	?	?	?	1	?	1	1
2	?	?	1	?	?	?	?	?	?	0	?	2
?	0	0	1	1	1	0	1	0	1	1	?	?
?	?	0	0	0	2	0	?	0	0	0	0	1
0	0	2	1	0	0	0	1	1	?	?	?	1
0	0	0	0	0	1	1	0	1	0	0	?	0
0	0	?	0	?	?	?	?	?	?	0	?	?
0	?	0	0	1	1	1	1	1	0	?	?	?
?	?	1	1	?	0	?	?	2	0	1	1	0
?	1	1	1	?	?	?	?	?	1	?	1	0
?	?	1	?	?	?	1	?	?	0	0	0	0
3	?	?	?	0	0	0	0	?				
Crocodylus megarhinus		?	?	?	?	?	?	0	?	?	?	?
?	?	?	?	?	?	?	0	0	1	?	?	?
?	?	?	?	?	?	?	?	?	?	?	?	?
?	?	?	?	?	?	?	?	?	1	1	1	0
2	1	0	1	?	?	?	?	?	0	0	1	2
0	0	0	0	?	?	0	1	0	1	1	?	?
?	?	0	0	1	1	0	?	0	0	0	0	?
0	0	2	3	0	0	0	0	1	0	0	0	1
?	0	0	0	0	?	?	0	?	0	0	0	0
0	0	1	1	0	1	0	0	0	1	0	1	2
0	0	0	0	1	1	1	1	1	0	?	0	0
2	?	1	1	?	0	0	1	2	?	0	1	0
0	1	0	1	0	?	0	1	1	0	0	?	?
?	1	1	1	0	0	1	0	0	0	0	0	0
3	?	?	?	0	0	0	0	?				
Australosuchus clarkae		?	?	?	?	?	?	0	?	?	?	?
?	?	?	1	?	?	0	?	?	1	?	?	?
?	?	1	1	?	?	?	?	?	?	?	0	?
1	0	?	?	?	1	?	?	?	1	1	1	0
?	1	0	1	?	?	?	?	?	0	0	1	2
0	0	0	0	1	1	0	1	0	1	1	?	?
?	?	0	0	1	1	0	?	0	0	0	0	1
1	0	2	1	0	0	0	0	1	0	0	0	1
0	0	0	0	0	1	?	0	?	0	0	0	0
0	0	?	?	?	?	?	?	?	1	0	1	0
0	0	?	0	1	1	1	1	1	0	?	0	0
2	0	1	1	?	0	1	1	2	0	0	1	0
0	1	0	1	0	0	0	1	1	?	?	1	?
?	1	1	?	0	0	1	0	0	0	?	0	0
1	?	?	?	0	0	0	0	?				
Kambara implexidens		?	?	?	?	?	?	0	?	?	?	?
?	?	?	?	?	?	?	?	0	1	?	?	?

	?	?	1	1	?	?	?	?	1	1	0	0	?
	1	0	?	?	?	1	?	?	?	1	1	1	0
	2	1	0	1	?	?	?	?	?	0	0	1	2
	0	0	0	0	1	1	0	1	0	1	1	?	?
	?	?	0	0	1	1	0	?	0	0	0	0	1
	1	0	2	1	0	0	0	0	1	0	0	0	1
	0	0	0	0	0	1	?	0	1	0	0	0	0
	0	0	1	0	0	1	0	0	0	1	0	1	0
	0	0	0	0	1	1	1	1	1	0	?	0	0
	2	0	1	1	?	0	0	1	2	0	0	1	0
	0	1	0	1	0	0	0	1	1	1	0	1	?
	1	1	1	1	0	0	1	0	0	0	0	0	0
	1	?	?	?	0	0	0	0	1				
Trilophosuchus rackhami				?	?	?	?	?	?	?	?	?	?
	?	?	?	?	?	?	?	?	?	?	?	?	?
	?	?	?	?	?	?	?	?	?	?	?	?	?
	?	?	?	?	?	?	?	?	?	?	?	?	?
	?	?	?	?	?	?	?	?	?	?	?	?	?
	?	?	?	?	?	?	?	?	?	?	?	?	?
	?	?	?	0	?	?	?	?	?	0	?	?	?
	?	?	?	2	?	0	?	?	0	?	?	0	?
	1	0	0	0	?	?	?	?	0	?	1	?	0
	0	0	0	0	1	0	1	0	?	?	?	0	1
	?	0	?	?	0	1	1	1	1	1	0	?	?
	1	2	0	1	1	1	0	1	1	2	1	0	1
	0	0	0	0	1	0	2	0	1	1	1	0	1
	?	1	1	1	?	0	?	1	0	0	0	?	0
	0	1	?	?	?	?	?	?	0	?			
Quinkana spp		?	?	?	?	?	?	?	?	?	?	?	?
	?	?	?	?	?	?	?	?	?	?	?	?	?
	?	?	?	?	?	?	?	?	?	?	?	?	?
	?	?	?	?	?	?	?	?	1	1	?	0	?
	?	?	1	?	?	?	?	?	?	?	?	?	?
	?	?	?	?	?	?	?	?	1	?	?	?	?
	?	2	1	0	1	0	?	0	0	0	0	?	1
	0	0/2	1/5	0	0	0/1	0	1	0	0	0	1	0
	0	0	0	0	?	?	?	?	0	?	0	?	?
	?	?	?	?	?	?	?	?	1	?	?	1	0
	0	?	0	1	1	1	?	1	0	?	0	0	2
	0	1	1	1	0	0	1	2	1	0	1	0	0
	1	0	1	0	2	?	1	1	1	0	1	?	1
	1	?	?	0	1	?	0	0	0	?	?	?	1
	?	?	?	?	?	?	?	?					

Tomistoma schlegelii		0	2	1	0	0	0	0	0	1	?	0
0	1	0	1	0	0	0	1	0	1	1	0	0
0	1	1	1	1	1	1	0	1	1	0	0	0
1	0	1	3	0	1	1	0	1	1	1	2	2
?	1	0	4	0	0	0	0	0	1	0	0	2
0	0	0	0	0	0	0	1	0	1	0	0	0
0	1	0	0	1	2	0	0	0	0	0	0	1
1	0	2	1	0	0	0	0	1	0	1	0	1
0	0	0	0	0	1	1	0	1	0	0	0	1
0	0	1	0	0	1	0	0	0	1	0	1	1
0	0	0	0	1	1	1	1	1	0	0	0	0
0	1	1	0	1	0	0	1	2	1	0	1	0
0	1	0	1	0	0	0	1	1	0	0	1	0
1	1	1	1	0	0	1	0	0	0	0	0	0
3	1	1	1	0	0	0	0	1				
Tomistoma lusitanica		?	?	?	?	?	?	0	?	?	?	?
?	?	?	?	?	?	?	?	0	1	?	?	?
?	?	?	?	?	?	?	?	?	?	?	?	?
1	0	?	?	?	1	?	?	?	1	?	?	2
?	?	?	4	?	?	?	?	?	0	0	?	2
0	0	0	0	?	?	0	1	0	1	?	?	?
?	?	0	0	1	2	0	?	0	0	0	0	?
1	0	2	1	?	0	0	0	1	0	1	?	1
0	0	0	0	0	1	1	0	?	0	0	0	0
1	0	1	0	0	1	0	0	0	1	0	1	0
0	0	0	0	1	1	1	1	1	0	?	0	0
0	1	?	1	?	1	0	1	2	1	0	1	0
0	1	0	1	0	0	0	1	1	0	0	1	?
?	1	1	1	0	0	1	0	0	0	0	0	0
3	?	?	?	0	0	0	0	1				
Tomistoma petrolica		?	?	?	?	?	?	?	?	?	?	?
?	?	?	?	?	?	?	?	?	?	?	?	?
?	?	?	1	?	?	?	?	?	?	?	?	?
?	?	?	?	?	?	?	?	?	?	?	?	?
?	?	0	4	?	?	?	?	?	?	0	0	2
0	?	0	?	0	1	?	1	?	1	0	?	?
?	?	0	0	?	?	?	?	?	?	?	?	?
?	?	2	?	0	0	?	0	?	?	0	?	?
?	0	0	0	0	1	?	?	?	0	?	0	0
1	0	1	0	0	1	?	?	?	?	?	?	1
?	0	0	?	0	?	1	1	1	0	?	0	?
2	?	0	?	?	0	?	?	2	0	0	?	0
0	0	0	1	0	0	?	?	?	?	?	?	?
?	?	1	?	?	?	1	?	?	?	?	0	0
3	?	?	?	0	0	0	0	?				

Toyotamaphimaea machikanensis 0 0 1 0 0 1 0 0 ?
? 1 1 1 1 1 1 0 0 1 0 1 1
0 0 0 1 1 1 1 1 1 0 1 1 0
0 ? 0 0 ? ? ? 1 ? ? ? ? ?
2 2 ? ? 0 4 ? ? ? ? ? 1 0
0 2 0 ? 0 0 0 0 ? 1 0 1 0
0 0 ? ? 0 0 1 2 0 ? 0 0 0
0 1 1 0 0 4 0 0 0 0 1 0 ?
? ? 0 0 0 0 0 ? ? ? ? 0 0
0 0 ? 0 1 0 0 1 0 0 0 0 ?
1 0 0 0 0 0 0 1 1 1 1 0 ?
? 0 0 ? 1 ? ? 1 ? ? 2 1 0
1 0 ? 1 0 1 0 ? ? ? ? ? ?
? ? ? ? 1 1 0 0 1 0 ? 0 0
0 0 3 ? ? ? 0 0 0 0 1

Penghusuchus pani 0 0 0 0 0 0 1 1 1 0 0
0 1 0 ? ? 1 0 1 0 1 ? ? 1
0 1 1 1 1 ? 1 ? 0 1 0 0 ?
0 0 ? ? ? 1 ? ? ? 1 ? 2 2
? 1 0 4 ? ? ? ? ? 0 0 ? 2
0 ? 0 0 0 1 0 1 ? 0 ? 0 0
? ? 0 ? ? ? ? ? 0 ? ? ? ?
? ? 2 5 0 0 0 0 1 ? 0 ? ?
0 0 0 0 0 1 1 1 1 0 0 0 0
1 0 0 0 0 1 0 1 0 0 0 1 1
0 2 0 0 0 1 1 1 0 0 ? ? ?
? ? ? ? 1 ? ? ? 2 ? ? ? ?
? 1 0 1 0 ? ? ? ? ? ? 0 ?
? 0 1 1 ? 0 1 0 0 1 0 0 0
3 ? ? ? 0 0 0 0 ?

Gavialosuchus eggenbergensis ? ? ? ? ? ? ? ? ? ?
? ? ? ? ? ? ? ? ? ? 1 ? ?
? ? ? ? ? ? ? ? ? ? ? ? ?
? ? ? ? ? ? ? ? ? ? ? ? ?
? ? ? ? ? ? ? ? ? ? ? ? ?
? ? ? ? ? ? ? ? ? ? ? ? ?
? ? ? 0 0 1 2 0 ? 0 0 0 0
? 1 0 2 4 0 0 0 0 1 0 0 ?
? 0 0 0 0 0 ? ? ? ? 0 0 ?
0 1 0 1 0 0 1 0 0 0 ? 0 1
2 0 0 0 ? 0 ? 1 1 1 0 ? 0
? ? ? ? ? ? 1 ? 1 2 1 0 1
0 ? 1 0 1 0 0 ? ? ? ? ? ?
? ? 1 1 ? ? ? 1 0 0 0 0 0
0 3 ? ? ? 0 0 0 0 ?

Paratomistoma courtii		?	?	?	?	?	?	?	?	?	?	?
?	?	?	?	?	?	?	?	?	?	?	?	?
?	?	?	?	?	?	?	?	?	?	?	?	?
?	?	?	?	?	?	?	?	?	?	?	?	?
?	?	?	?	?	?	?	?	?	0	?	?	2
0	?	0	0	0	0	0	1	0	1	0	?	?
?	?	0	0	?	?	?	?	?	?	?	?	?
?	?	2	?	?	0	?	0	?	?	?	?	?
?	?	0	0	0	1	?	0	?	?	?	?	?
?	?	?	?	?	?	?	?	?	?	?	?	0
0	0	0	?	1	?	?	1	1	?	?	?	?
?	1	?	?	?	1	?	?	2	0	0	1	0
0	0	0	1	0	0	0	?	1	0	0	0	?
1	0	1	?	0	?	1	0	0	?	?	0	0
?	?	?	?	?	?	?	0	?				
Megadontosuchus arduini			?	?	?	?	?	?	?	?	?	?
?	?	?	?	?	?	?	?	?	0	1	?	?
?	?	?	?	?	?	?	?	?	?	?	?	?
?	1	?	?	?	?	?	?	?	?	1	1	2
0	?	?	?	?	?	?	?	?	?	0	?	?
2	0	?	?	?	?	?	0	?	?	?	1	?
?	?	?	0	0	1	?	0	?	0	0	0	?
1	1	0	2	1	0	0	0	0	1	?	?	?
?	?	?	0	0	0	?	?	?	2	0	?	?
?	1	?	?	?	?	?	?	?	?	?	?	?
?	0	0	0	?	0	1	1	1	1	0	?	?
?	0	?	?	?	?	1	?	?	2	0	0	1
?	?	1	0	1	?	?	?	?	?	?	?	?
?	?	?	1	?	?	?	1	?	?	?	?	0
0	?	?	?	?	0	0	0	0	?			
Tomistoma cairense		?	?	?	?	?	?	?	?	?	?	?
?	?	?	?	?	?	?	?	?	?	?	?	?
?	?	?	?	?	?	?	?	?	?	?	?	?
?	?	?	?	?	?	?	?	?	1	?	?	2
?	?	?	4	?	?	?	?	?	?	?	?	2
0	0	0	0	0	1	?	1	0	1	0	?	?
?	?	0	0	?	2	0	?	0	?	?	?	?
?	0	2	5	?	0	?	?	1	?	0	?	?
?	?	0	0	0	?	?	0	?	0	0	0	0
1	0	1	0	0	1	0	0	0	1	0	?	0
0	?	0	?	0	1	1	1	1	0	?	?	?
0	1	?	?	?	1	0	?	2	0	0	1	0
0	1	0	1	0	0	0	1	?	?	0	?	?
?	1	1	?	0	0	1	?	0	0	0	0	0
3	?	?	?	0	0	0	0	?				

Thecachampsa antiqua 0 2 0 0 0 0 0 0 ? ? 0
0 1 0 1 0 ? 0 0 0 1 1 0 1
0 1 1 1 ? 1 ? ? 1 1 ? 0 ?
0 0 ? ? ? 1 ? ? ? 1 1 2 2
? ? ? 4 ? 1 0 0 ? ? ? ? 2
0 0 0 0 3 1 ? 1 0 1 ? 0 ?
? ? 0 0 1 2 0 ? 0 0 0 0 1
1 0 2 1 0 0 0 0 1 0 0 ? 1
0 0 0 0 0 ? ? 0 ? 0 0 0 0
1 0 1 ? 0 1 0 0 0 1 0 1 1
0 0 0 0 0 1 1 1 1 0 ? ? ?
0 1 1 ? ? 1 0 1 2 0 0 1 0
0 1 0 1 0 1 0 ? ? 0 0 ? ?
? 1 1 1 0 0 1 0 0 ? 2 0 0
3 ? ? ? 0 0 0 0 1

Thecachampsa americana ? ? ? ? ? ? ? ? ? ?
? ? ? ? 1 ? ? ? ? 0 1 ? 0
0 0 ? 1 1 1 1 ? ? 1 1 0 0
? 0 0 ? ? ? 1 ? ? ? 1 1 2
2 ? ? ? 4 ? ? ? ? ? 0 0 0
2 0 ? 0 0 3 1 ? 1 0 1 0 ?
? ? ? 0 0 1 2 0 ? 0 0 0 0
1 1 0 2 1 0 0 0 0 1 0 0 ?
1 0 0 0 0 0 ? ? ? ? 0 0 0
0 1 0 0 0 0 1 0 0 0 ? ? ?
1 0 0 0 ? 0 1 1 1 1 0 ? 0
0 0 ? 1 0 ? 1 0 1 2 0 0 1
0 0 1 0 1 0 1 ? ? ? 0 ? ?
? ? 1 1 1 0 0 1 0 0 ? 2 0
0 3 ? ? ? 0 0 0 0 1

Thecachampsa carolinense ? ? ? ? 0 0 0 0 ? ?
? ? ? 0 1 0 ? 0 1 0 1 1 0
? 0 1 ? ? ? ? ? ? 1 ? 0 0
? 0 0 ? ? ? 1 ? ? ? 1 1 2
2 ? ? 0 4 ? ? ? ? ? 0 0 0
2 0 ? 0 1 3 1 ? 1 0 1 0 ?
? ? ? 0 0 1 2 0 ? 0 0 0 0
1 1 0 2 1 0 0 0 0 1 ? ? ?
? 0 0 0 0 0 ? ? ? ? 0 ? 0
0 1 0 1 0 0 1 0 0 0 ? 0 1
? 0 0 0 ? 0 ? 1 1 1 0 ? ?
? 0 ? ? ? ? 1 ? ? 2 0 0 1
0 ? 1 0 1 0 1 ? ? ? ? ? ?
? ? ? 1 1 0 0 1 0 0 ? 2 0
0 3 ? ? ? 0 0 0 0 ?

Dollosuchoides densmorei 0 0 1 ? ? ? 0 ? ? ?
1 1 1 0 1 0 ? 0 0 0 1 ? 0
? 0 0 1 1 1 1 ? ? 1 ? 0 0
? ? ? ? ? ? ? ? ? ? 1 1 2
0 ? ? ? 0 ? ? ? ? ? 0 0 0
2 0 0 ? 0 ? ? 0 1 0 1 ? ?
? ? ? 0 0 1 2 0 ? 0 0 ? 0
? 1 0 2 1 0 0 0 0 1 0 0 ?
? 0 0 0 0 0 ? ? ? ? 0 1 ?
0 1 0 1 0 0 1 0 0 0 1 0 1
1 0 0 0 ? 0 1 1 1 1 0 ? ?
? ? ? ? ? ? 1 ? ? 2 0 0 1
0 ? 1 0 1 0 0 0 1 ? ? ? ?
? ? ? 1 0 ? 0 1 0 0 0 ? 0
0 3 ? ? ? 0 0 0 0 1

Kentisuchus spenceri ? ? ? ? ? ? 0 ? ? ? ?
? ? ? ? ? ? ? ? ? ? ? ? ?
? ? ? ? ? ? ? ? ? ? ? ? ?
? ? ? ? ? ? ? ? ? ? 1 ? ?
? ? ? 0 ? ? ? ? ? 0 ? ? 2
0 0 0 ? 1 1 0 1 ? 1 1 ? ?
? ? 0 0 1 1 0 ? 0 0 0 0 ?
1 0 2 1 0 0 0 0 1 0 0 ? ?
? 0 0 0 0 ? ? 0 ? 0 1 0 0
1 0 1 0 0 1 0 0 ? 1 0 1 1
0 0 0 ? 1 1 1 1 1 0 ? ? ?
? ? ? 0 ? 0 ? ? 2 0 0 1 0
? 1 0 1 0 0 ? ? ? ? ? ? ?
1 1 1 ? 0 ? 1 0 0 ? 0 ? 0
3 ? ? ? 0 0 0 0 ?

Brachyuranochampsa eversolei ? ? ? ? ? ? ? ? ? ?
? ? ? ? ? ? ? ? ? ? ? ? ?
? ? ? ? ? ? ? ? ? ? ? ? ?
? ? ? ? ? ? ? ? ? ? ? ? ?
? ? ? ? ? ? ? ? ? ? ? ? ?
? ? ? ? ? ? ? ? ? ? ? ? ?
? ? ? 0 0 1 1 ? ? 0 0 ? ?
? 1 0 2 1 0 0 0 0 1 0 0 ?
? 0 0 0 0 0 ? ? 0 ? 0 ? 0
? ? 0 1 1 0 1 0 0 0 1 0 1
0 0 0 1 ? 1 1 1 1 0 0 ? ?
? 2 ? 0 0 ? 0 ? 1 2 1 0 1
0 ? 1 0 1 0 0 0 ? ? ? ? ?
? ? 1 1 0 0 0 1 0 0 0 0 0
0 3 ? ? ? 0 0 0 0 ?

Crocodylus acer ? ? ? ? ? ? ? ? ? ? ?
? ? ? ? ? ? ? ? ? ? ? ? ?
? ? ? ? ? ? ? ? ? ? ? ? ?
? ? ? ? ? ? ? ? ? ? ? ? ?
? ? ? ? ? ? ? ? ? ? ? ? ?
? ? ? ? ? ? ? ? ? ? ? ? ?
? ? 0 0 1 1 0 ? 0 0 0 0 ?
1 0 2 1 0 0 0 0 1 0 0 ? ?
0 0 0 0 0 1 ? ? ? 0 0 0 1
0 0 ? 1 0 1 0 0 0 1 0 1 0
0 0 1 0 1 1 1 1 0 0 ? 0 0
2 ? 0 1 ? 0 0 ? 2 0 0 1 0
0 1 0 1 0 0 0 1 ? ? 0 ? ?
1 1 1 0 0 0 1 0 0 0 0 0 0
3 ? ? ? ? 0 0 0 ?

Asiatosuchus grangeri ? ? ? ? ? ? ? ? ? ? ?
? ? ? ? ? ? ? ? ? ? ? ? ?
? ? ? ? ? ? ? ? ? ? ? ? ?
? ? ? ? ? ? ? ? ? 1 ? ? 0
? 1 0 1 ? ? ? ? ? ? 0 ? 2
0 0 0 ? ? ? ? 1 0 1 ? ? ?
? ? 0 ? ? ? 0 ? ? ? ? ? ?
? ? 1 ? ? 0 ? ? ? ? ? ? ?
1 ? ? ? ? ? ? ? ? ? ? ? ?
? ? ? ? ? ? ? ? ? ? ? ? ?
0 0 0 ? ? ? 1 1 0 ? ? ? ?
? ? ? ? ? ? ? ? 2 0 ? ? ?
? ? 0 ? ? ? ? ? ? ? ? ? ?
? ? ? ? ? ? ? ? ? ? ? ? ?
? ? ? ? ? ? ? 0 ?

Crocodylus depressifrons ? ? ? 0 0 ? 0 0 ? ?
1 1 0 0 0 0 1 0 0 1 1 1 0
0 0 0 1 1 1 1 ? ? 1 1 0 0
? 1 0 ? ? ? 1 ? ? ? 1 1 1
0 2 1 0 1 ? ? ? ? ? 0 0 1
2 0 0 0 0 0 0 0 1 0 1 1 ?
? ? ? 0 0 1 1 0 ? 0 0 0 0
? 1 0 1 1 0 0 0 0 1 0 0 ?
1 1 0 0 0 0 1 ? 0 ? 0 0 0
1 0 0 1 ? 0 1 0 0 0 1 0 1
? 0 0 0 0 1 1 1 1 0 0 ? 0
1 0 2 0 0 ? 0 0 1 2 0 0 1
0 0 1 0 1 0 0 0 1 1 0 0 1
? 1 1 1 0 0 0 1 0 0 0 0 0
0 3 ? ? ? 0 0 0 0 1

Crocodylus affinis 0 0 1 0 0 1 0 0 1 ? 1
0 0 0 1 0 0 0 0 1 1 1 0 0
0 0 1 1 1 1 0 0 1 1 0 0 ?
1 0 ? ? ? 1 ? ? ? 1 1 1 0
2 1 0 1 0 1 0 0 0 0 0 1 2
0 0 0 0 0 0 0 1 0 1 1 0 0
? ? 0 0 1 1 0 ? 0 0 0 0 1
0 0 1 1 0 0 0 0 1 0 0 ? 0
1 0 0 0 0 ? ? 0 ? 0 0 0 1
0 0 1 1 0 1 0 0 0 1 0 1 0
0 0 0 0 1 1 1 1 0 0 ? 0 ?
0 1 0 ? ? 0 0 1 2 0 0 1 0
0 1 0 1 0 0 0 1 ? ? 0 ? ?
? 1 1 0 0 0 1 0 0 0 0 0 0
3 ? ? ? 0 0 0 0 1

Asiatosuchus germanicus 0 0 1 ? 0 ? 0 ? 1 ?
0 0 1 0 1 0 ? 0 1 0 1 ? 0
0 0 ? 1 1 1 1 ? ? 1 ? ? 0
? ? ? ? ? ? 1 ? ? ? 1 1 1
0 2 0 0 0 ? ? ? ? ? 0 0 1
2 0 0 0 0 ? ? 0 1 0 1 ? 0
0 ? ? 0 0 0 1 0 ? 0 0 0 0
? 0 0 0 1 0 0 0 0 1 0 ? ?
? 1 0 0 0 0 ? ? 0 ? 0 0 0
1 0 ? ? ? 0 1 0 0 0 1 0 1
0 0 0 0 ? 1 1 1 1 0 0 ? ?
? 0 1 0 0 ? 0 0 1 1 1 0 1
0 0 1 0 1 0 0 0 ? ? ? ? ?
? ? 1 1 0 0 ? 1 0 0 0 0 0
0 3 ? ? ? 0 0 0 0 1

Prodiplocynodon langi ? ? ? ? ? ? ? ? ? ? ?
? ? ? ? ? ? ? ? ? ? ? ? ?
? ? ? ? ? ? ? ? ? ? ? ? ?
? ? ? ? ? ? ? ? ? ? ? ? ?
? ? ? ? ? ? ? ? ? ? ? ? ?
? ? ? ? ? ? ? ? ? ? ? ? ?
? ? 0 0 1 1 0 ? 0 0 0 0 ?
? 0 0 3 0 0 0 0 1 0 0 ? ?
1 0 0 0 0 1 ? ? 0 0 0 0 1
0 0 1 1 ? 1 0 0 0 1 0 1 ?
? ? 0 ? 1 1 1 1 0 0 ? ? ?
0 ? 0 ? ? 0 ? 1 1 ? 0 1 0
? 1 0 1 0 0 0 1 ? ? 0 1 ?
0 1 1 0 0 1 1 0 0 0 0 0 0
3 ? ? ? 0 0 0 0 ?

Crocodylus checchiai MPURLS LIBYA				?	?	?	?	?	?	?	?	?
?	?	?	?	?	?	?	?	?	?	?	?	?
?	?	?	?	?	?	?	?	?	?	?	?	?
?	?	?	?	?	?	?	?	?	?	?	?	?
?	?	?	?	?	?	?	?	?	?	?	?	?
?	?	?	?	?	?	?	?	?	?	?	?	?
?	?	?	?	0	0	1	1	0	?	0	0	0
0	1	0	0	2	1	0	1	0	0	1	0	0
?	1	0	0	0	0	0	1	1	0	1	0	0
1	0	0	0	1	1	0	1	0	0	0	?	0
?	0	0	0	0	0	1	1	1	1	1	0	?
?	?	1	0	1	0	1	0	0	1	2	0	0
1	0	0	1	0	1	0	0	?	1	1	1	0
1	0	1	1	?	?	0	0	1	1	0	0	0
0	0	3	?	?	?	0	0	?	0	?		
