# Supplementary material for: Old African fossils provide new evidence for the origin of the American crocodiles
Source: Sci Rep. 2020 Jul 23;10:11127. doi: 10.1038/s41598-020-68482-5 (PMC7378212; doi:10.1038/s41598-020-68482-5)
Supplement: Supplementary file 2 — Supplementary Information 2. [file 41598_2020_68482_MOESM2_ESM.pdf]

Supplementary Data File

Old African fossils provide new evidence for the origin of the American crocodiles

Massimo Delfino, Dawid A. Iurino, Bruno Mercurio, Paolo Piras, Lorenzo Rook & Raffaele Sardella

Matrix Scheyer et al with *C. checchiai* Libya

Crocodylus megarhinus ? ? ? ? ? ? ? 0 ? ? ?
? 1 0 0 0 0 0 0 1 0 2 1 0
0 ? 1 0 0 ? 0 0

Crocodylus pigotti ? ? 0 0 ? ? 0 0 1 1 ?
? 1 ? 0 1 0 1 1 1 1 ? ? 0
1 0 ? 1 0 0 1 1

Rimasuchus lloydi ? ? ? ? ? ? ? ? ? ? ?
? ? ? 0 1 ? 0 0 ? ? 1 1 0
1 0 1 1 0 ? 0 0

Voay robustus ? ? ? ? ? ? ? ? ? ? 1 1
1 0 0 1 0 0 0 1 2 0 1 1 1
1 ? 1 0 0 0 1

Mecistops cataphractus 1 0 0 0 0 0 0 0 1 1
2 0 1 1 0 0 0 0 1 1 1 0 1
0 0 0 1 1 0 0 1 0

Crocodylus palaeindicus ? ? ? ? ? ? ? ? ? ?
? ? 0 1 0 0 0 1 0 0 1 0 1
0 0 1 0 1 1 ? 1 ?

Crocodylus porosus 1 1 0 0 1 1 0 1 0 1 2
0 0 1 0 1 1 0/1 0 1 1 0 1 0
0 0 1 1 1 1 0 1

Crocodylus palustris 1 0 0 0 1 0 1 1 1 1 2
1 0 1 0 1 1 0/1 0 1 1 0 1 0
0 0 1 1 1 1 0 1

Crocodylus siamensis 1 1 1 1 1 0 0 0 1 0 2
0 0 1 0 1 1 0/1 0 1 1 0 1 1
0 0 1 1 1 1 0 0

Crocodylus johnstoni 1 1 0 0 1 1 0 1 0 1 2
0 ? 1 0 1 1 0/1 1 1 0 0 1 0
0 0 1 1 1 0 0 0

Crocodylus mindorensis 1 1 0 0 1 1 0 1 0 1
2 0 0 1 0 1 1 0/1 0 1 1 0 1
0 0 0 1 1 1 1 1 0

Crocodylus novaeguineae 1 1 0 0 1 1 0 1 0 1
2 0 0 1 0 1 1 0/1 0 1 1 0 0
0 0 0 1 0 1 0 0 0

Crocodylus niloticus 1 0 1 0 1 0 0 1 1 1 2
0 0 1 0 0 1 0/1 0 1 1 1 1 0
0 0 1 1 1 0 0 0

Crocodylus falconensis ? ? ? ? ? ? ? ? ? ? ?
? 0 1 1 0 1 0 0 0 ? 1 ? 0
0 ? ? ? ? 0 ? 0

Crocodylus acutus 0 0 1 0 1 0 0 1 1 1 2
0 0 1 1 0 1 0/1 0 0 1 1 1 0
0 0 1 1 1 1 1 1

Crocodylus intermedius0 0 1 0 1 0 0 1 1 1 2
0 1 1 1 0 1 0/1 0 0 1 0 1 0
0 0 1 1 1 1 1 1

Crocodylus moreletii 0 0 1 0 1 0 0 1 1 1 2
0 0 1 1 0 1 0/1 0 0 1 1 1 0
0 0 1 1 1 1 1 0

Crocodylus rhombifer 0 0 1 0 1 0 0 1 1 1 1
0 0 1 1 0 1 0/1 0 0 1 1 1 1
0 0 1 1 1 1 1 0

Crocodylus checchiai LIBYA ? ? ? ? ? ? ? ? ? ?
? ? ? ? 1 0 ? 1 0 1 1 0 0
0 0 0 1 1 1 1 1 0
