# Supplementary material for: Old African fossils provide new evidence for the origin of the American crocodiles
Source: Sci Rep. 2020 Jul 23;10:11127. doi: 10.1038/s41598-020-68482-5 (PMC7378212; doi:10.1038/s41598-020-68482-5)
Supplement: Supplementary file 4 — Supplementary Information 4. [file 41598_2020_68482_MOESM4_ESM.docx]

**Supplementary information**

Old African fossils provide new evidence for the origin of the American crocodiles

Massimo Delfino, Dawid A. Iurino, Bruno Mercurio, Paolo Piras, Lorenzo Rook & Raffaele Sardella

**Supplementary Note 1: Comparison between the character coding of the Libyan and Tanzanian materials**

The coding of the Tanzanian *C. checchiai* differs from the one we propose here for the Lybian specimen for 41 characters. Of these, two characters (82, the morphology of anterior process of the ectoperygoids and, 114, the relationship between the nasals and the external naris) were scored differently in the two matrixes and have been commented in the main text. Some of the 29 characters scored by Brochu and Storrs^9^ and not by us are related to postcranial elements that were not available to us, others related to cranial structures that are not well preserved in the Libyan skull (so that they were not scorable with confidence). Preservational reasons are very likely responsible for the 10 characters that we scored by us for the Libyan skull but were not scored by Brochu and Storrs^9^ for the Tanzanian material. In some cases, the CT data allowed us to confidently interpret some morphological traits. Worth underlining is that the fact that the Libyan skull redescribed by us and the Tanzanian material differ for only two characters out of 189 (all the others being congruent or not overlapping because scored only for one of them), and the fact that one of them appears to be variable in different skulls from Libya, do not allow us to express general considerations against the conspecificity of the Libyan and the Tanzanian crocodiles characterized by a with a medial rostral boss. In fact, the phylogenetic analysis, see below, does not help to clarify this issue.

**Supplementary Note 2: Notes on the interrelationships among the extant American crocodiles**

As far as the extant American crocodiles are concerned, the topology we obtained in both trees is different from those previously published by other authors, topologies that were nevertheless in contrast with each other (compare for example Hekkala et al.^4^ with Oaks^2^). In both our analyses (see Fig. 4 and 5), *C. intermedius* has a basal position in respect to the other three extant American species and it is not the sister taxon of *C. acutus*^2,4^. However, the recent analysis of the mitochondrial genome by Milián-García et al.^[1]^ concluded that there are two, previously undetected, lineages of *C. acutus*, one clustering with *C. intermedius* and one with *C. rhombifer*. The knowledge of the phylogenetic relationships of the American extant crocodiles is therefore still in a state of flux, and since it is not known which is the origin of the specimen(/s) of *C. acutus* on which the morphological coding we used was prepared (Brochu & Storrs^9^), nor if there are osteological differences among the recently detected two clades of *C. acutus*, it is not possible to further discuss on a firm ground the results of our analyses.

**Supplementary Note 3.**

Character coding of *Crocodylus checchiai* based sn813/lj on the basis of the character list by Brochu & Storrs^9^:

?????????? ?????????? ?????????? ?????????? ?????????? ?????????? ?????????? ????????00 110?000010 0210100100 ?100000110 1001000110 1000?0?000 00111110?? ?101010012 0010010100 ?11101011? ?001100000 3???00?0?

**Supplementary Note 4. Phylogenetic analysis based on Brochu & Storrs (2012) including both the codings of *C. checchiai***

We included our coding of *C. checchiai* based on the topotypic Libyan specimen sn813/lj in the matrix by Brochu & Storrs^9^ without removing the coding of the same species based on specimens from the late Miocene Nawata Formation in the western Lake Turkana Basin (see Supplementary Data File 3). The position of the Libyan *C. checchiai* is therefore tested along with that of the Tanzanian material referred by Brochu & Storrs^9^ to the same species. We obtained a topology (Supplementary Fig. S1) that broadly mirrors that of Brochu & Storrs^9^ and shows a broad polytomy that includes both the *C. checchiai* codings along with several extant taxa (*C. niloticus*, *C. palustris*, *C. siamensis*, *C. intermedius*), the extinct *C. palaeindicus* and three clades of Crocodylus species (two with only extant and one with only extinct taxa) that have a biogeographic and phylogenetic congruence. It therefore not possible to retrieve the two codings of *C. checchiai* in a clade.


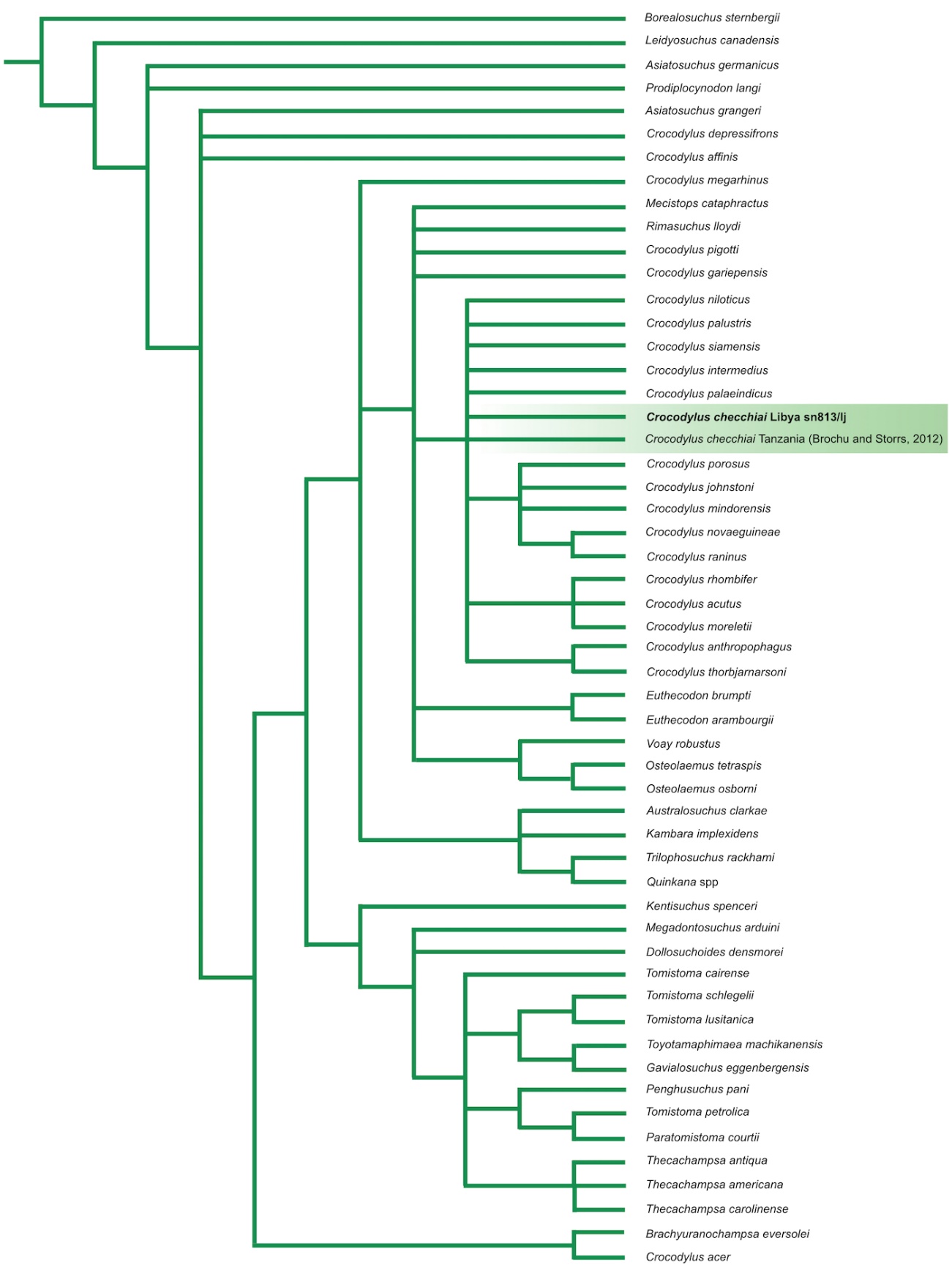


**Supplementary Fig. S1.** Strict consensus of 6 equally parsimonious trees with 313 steps and an adjusted homoplasy which ranges between 29.2 and 29.7, Consistency Index is 0.473, and Retention index is 0.722. The strict consensus tree is 342 steps long with an adjusted homoplasy of 32.7, Consistency Index is 0.433, and Retention index is 0.673.

**Supplementary Note 5.**

Character coding of *Crocodylus checchiai* based sn813/lj on the basis of the character list by Scheyer et al.^19^:

?????????? ????10?101 1000001111 10

**Supplementary References**

**[1].** Milián-García Y, Russello MA, Castellanos-Labarcena J, Cichon M, Kumar V, Espinosa G, Rossi N, Mazzotti F, Hekkala E, Amato G, Janke A. Genetic evidence supports a distinct lineage of American crocodile (Crocodylus acutus) in the Greater Antilles. *PeerJ.* **6**, e5836 (2018).
